# Investigating the antidepressant effect of Ziyan green tea on chronic unpredictable mild stress mice through fecal metabolomics

**DOI:** 10.3389/fmicb.2023.1256142

**Published:** 2023-08-24

**Authors:** Wenbao Jia, Qian Tang, Yao Zou, Yang Yang, Wenliang Wu, Wei Xu

**Affiliations:** ^1^College of Horticulture, Tea Refining and Innovation Key Laboratory of Sichuan Province, Sichuan Agricultural University, Chengdu, China; ^2^Tea Research Institute, Hunan Academy of Agricultural Sciences, Changsha, Hunan, China; ^3^Sichuan Yizhichun Tea Industry Co., Ltd., Muchuan, Sichuan, China

**Keywords:** Ziyan green tea, CUMS, depression-like behaviors, neurochemical factors, pro-inflammatory factors, gut microbiota, fecal metabolites

## Abstract

**Introduction:**

Some studies have shown the effectiveness of tea in reducing depression. Gut flora dysfunction is strongly associated with depression. The mechanism by which Ziyan green tea ameliorates depression is not clear.

**Methods:**

In this study, we examined the impact of Ziyan green tea on mice exhibiting symptoms similar to depression. We specifically focused on the role of intestinal flora and its metabolites. We first established a chronic unpredictable mild stress (CUMS) mouse model to induce depressive symptoms and conducted behavioural tests, biochemical tests, and pathological tissue analysis. We also investigated gut microbiota changes by 16S rRNA sequencing and measured faecal metabolites in mice using UHPLC-MS/MS.

**Results:**

The results showed that Ziyan green tea intervention improved depression-like behaviour, neurobiochemical factors, and reduced levels of pro-inflammatory factors in CUMS mice. Spearman’s correlation analysis showed that different microbial communities (*Corynebacterium*, *Faecalibaculum*, *Enterorhabdus*, *Desulfovibrio*) correlation with differential metabolites (Cholic acid, Deoxycholic acid, etc.) and depression-related biochemical indicators (5-HT, DA, BDNF, IL-6, and TNF-α).

**Discussion:**

In conclusion, our findings suggest that both low and high-dose interventions of Ziyan green tea have positive preventive effects on CUMS mice without dose dependence, partly because they mainly affect intestinal Purine Metabolism, Bile Acid Biosynthesis and Cysteine Metabolism in CUMS mice, thus stimulating brain 5-HT, DA and BDNF, and decreasing the inflammatory factors IL-6, TNF-α, activate the composition of intestinal flora, improve the intestinal flora environment and thus promote the production of intestinal metabolites, which can be used for depression treatment. It is suggested that Ziyan green tea may achieve an antidepressant effect through the gut-microbiota-brain axis.

## 1. Introduction

Depression, as a chronic illness, poses a significant threat to human well-being, manifesting through enduring feelings of low mood, cognitive deceleration, diminished interest, heightened anxiety, and various additional symptoms (Kandola et al., 2019). It is a highly prevalent global psychiatric disorder, the incidence of which has been increasing in recent years and has become a common psychological disorder (Lach et al., 2018). Research findings indicated that around 4.7% of the global populace encounters episodes of depression within a span of 12 months (Herrman et al., 2022). Presently, the clinical management of individuals suffering from depression is predominantly centered around the administration of antidepressant medications, such as Selective Serotonin Reuptake Inhibitors (SSRIs), Tricyclic Antidepressive Agents (TCAs), Monoamine Oxidase Inhibitors (MAOIs) and Noradrenergic and specific serotonergic antidepressants (NaSSAs). Nevertheless, these aforementioned categories of antidepressants are associated with various undesirable consequences, encompassing insomnia, diarrhea, headaches, and other adverse effects (Zhang et al., 2017; Chow and Issa, 2019). These adverse effects limit the use of drugs in the treatment of depression. Therefore, there is a need to find other alternatives that are biologically active and have fewer side effects than antidepressants or have preventive effects.

It has been shown that tea can reduce the likelihood of subthreshold depression and prevent the worsening of existing symptoms (Ng et al., 2021). Green tea catechins have been shown to alleviate depressive symptoms in experimental animals, possibly through the inhibition of monoamine oxidase (Nabavi et al., 2017). Studies suggested that tea polyphenols may have antidepressant properties and may reduce depression risk and severity in humans through regular consumption of tea (Siddiqui et al., 2004; Zhu et al., 2012; Pervin et al., 2018; de la Garza et al., 2019; Rothenberg and Zhang, 2019), among older Chinese people, regular consumption of green tea is unlikely to result in depressive symptoms (Yao et al., 2021). Additionally, the study found that regular green tea consumption (≥3 cups/week) was negatively associated with self-reported lifetime depression among Korean adults, suggested that the prevalence of depression was 21% lower among regular green drinkers than among non-green tea drinkers (Zhu et al., 2012). It is believed that tea extracts act as antidepressants by enhancing intestinal flora, increasing the content and diversity of beneficial bacteria, and inhibiting the growth of harmful bacteria (Shao et al., 2022). In addition, related studies have reported that anthocyanins have antidepressant effects, and some studies have used extracts such as blueberry and *vaccinium myrtillus*, which have high anthocyanin content. Blueberry extract has antidepressant effects and protects the brain from oxidative damage (Spohr et al., 2022). Krikorian et al. (2010) used blueberry juice to continuously intervene in the elderly with mild cognitive impairment for 12 weeks. As a result, while the cognitive function of these elderly people was significantly improved, their depressive symptoms also tended to improve. In addition, Kumar et al. (2012) used low, medium, and high doses of *vaccinium myrtillus* extract to intervene in depression model mice caused by chronic stress. The immobility time of the animals in the 500 mg/kg dose group was significantly reduced, and the number of spontaneous activities of the animals in the 500 mg/kg dose group was increased. The sugar water preference of the animals in the 250 mg/kg and 500 mg/kg dose groups was significantly increased, indicating that the anthocyanin-rich bilberry extract has an antidepressant effect. The new variety of purple buds with high anthocyanin, Ziyan, has been shown to be suitable for green tea (Chunjing et al., 2020), and the preventive effect of Ziyan green tea on depression is unknown.

A dysfunctional or damaged neurotransmission system in the brain, specifically involving 5-HT and BDNF, are closely associated with depression (Zhu et al., 2012). Bioactive compounds in tea increase levels of monoamine neurotransmitters, such as 5-HT and DA, which regulate depression. Research has shown that depression is associated with insufficient concentrations of 5-HT in the brain gaps, leading to mental disorders. It is associated with an overall decrease in activity and mental functioning (Zhang et al., 2017). Among the effects of inflammation on depression are changes in lipopolysaccharide, inflammatory cytokines, presynaptic neurotransmitter reuptake, microglial activation, and HPA axis activity. The serum levels of proinflammatory molecules such as IL-6 and TNF-α are elevated in depressed patients (Köhler et al., 2017).

Microbiota-gut-brain (MGB) axis mediates two-way communication between the brain and gut (Doroszkiewicz et al., 2021), which plays an important role in depression (Lucidi et al., 2021). Cerebral ischemia rapidly causes intestinal ischemia, excessive nitrate is increased through free radical reaction, and the increase of enterobacteria leads to intestinal flora imbalance. The expansion of enterobacteria can aggravate systemic inflammation and cerebral infarction (Xu et al., 2021). An altered gut microbiota composition leads to altered gut barrier permeability, monoamine neurotransmitter levels, hypothalamic–pituitary–adrenal (HPA) axis activity, and brain-derived neurotransmitters (Liu et al., 2021). Existing research suggested that changes in the gut microbiome may distinguish patients with MDD from healthy individuals. Increased levels of pro-inflammatory cytokines associated with social avoidance are influenced by *Lachnospiraceae* (Rosa et al., 2022). The decline in gut bacterial diversity can affect the stability of the microbiota, resulting in increased dominance of potentially harmful bacteria, decreased beneficial bacterial genera, and decreased microbial richness and diversity (Shao et al., 2022).

Moreover, the metabolites of gut microbiota play an important role in gut-brain communication (Lourenço et al., 2019), it has been shown that microbe-derived compounds such as bile acids might contribute to psychiatric disorders via the gut-microbiota-brain axis (Chang et al., 2022; Hashimoto, 2022). Dysbiosis of gut microbiota is associated with systemic inflammation because bile acids are a major regulator of the gut microbiota. As a whole, it is believed that the bile acid-gut-microbiota axis plays a role in immune regulation and health (Ridlon et al., 2014).

In this study, we investigated the mechanisms and effects of Ziyan green tea on CUMS-induced depression by using behavioral tests, neurobiochemical factor levels, pro-inflammatory factor levels, and pathological tissue analysis, and measured and analyzed the intestinal microbial diversity, abundance, and related metabolites in CUMS mice. Our study provides new insights into the antidepressant mechanism of Ziyan green tea.

## 2. Materials and methods

### 2.1. Plant material and HPLC analysis of tea water extract

The raw materials of the experiment were Ziyan [*Camellia sinensis* (L.)], and Ziyan green tea was made with one bud and two leaves according to the GB/T 14456.3-2016. The preparation of Ziyan green tea extract was referred to the study by Wenliang et al. (2018). Take an appropriate amount of Ziyan green tea samples, according to the material-liquid ratio of 1:10, steep extraction 3 times (boiling water), each extraction time of 20 min, gauze filtration, combined extraction solution, concentrated to a certain volume under reduced pressure, pre-freeze at −20°C for 12 h, put into the freeze dryer for 24 h, collect tea powder, sealed packaging, −20°C storage.

The chemical composition of Ziyan green tea aqueous extracts was analyzed using our previous method (Jia et al., 2022). Briefly, the contents of tea polyphenols (TPs), free amino acids (AA), soluble sugars (SS), Theaflavins (TFs), and Thearubigins (TRs) were determined using spectrophotometry. Total catechins (DL-C, catechin; EC, epicatechin; EGC, epigallocatechin; ECG, epicatechin gallate; GCG, gallocatechin gallate; EGCG, epigallocatechin gallate; CG, catechin gallate; GC, gallocatechin), Theobromine (TB), Theophylline (THEO), Caffeine (CAF) contents of the extracts were determined using high performance liquid chromatography (HPLC). The anthocyanin content was determined by the PH difference-in-difference method (Lee et al., 2005).

### 2.2. Animals and experimental design

C57BL/6 J male mice (20 ± 2 g) were used for this study. Mice were procured from Hunan Slake Jingda Experimental Animal Co., Ltd., animal qualification certificate number SYXK (Xiang) 2020–0008. The animals were housed at 22 ± 2°C and a relative humidity of 55 ± 5 with a 12:12 h light and dark cycle. The animals were acclimatized for a period of two weeks before the study. All operations were performed in accordance with the “Guide for the Care and Use of Laboratory Animals (8th Edition)” issued by the Animal Ethics Committee of the Hunan Provincial Center for Drug Safety Evaluation and Research.

After their acclimatization, the mice were divided randomly into four groups: control group (Con, *n* = 10), CUMS model group (Mod, *n* = 10), CUMS mice treated with Ziyan green tea soup at a low dose (ZY-L, *n* = 10), and CUMS-exposed mice treated with Ziyan green tea soup at a high dose (ZY-H, *n* = 10). The dose of gavage was based on a previous study (Wenliang et al., 2018), to put it simply, if an adult (average body weight 60 kg) drinks 10 g of tea per day, i.e., 166.67 mg·kg^−1^·d^−1^, then the dose of tea for mice should be 1,516.67 mg·kg^−1^·d^−1^. The dose of tea powder given = 166.67 × Mulriple × Extraction rate (freeze-dried powder of tea extract, per day), The extraction rate of tea was calculated according to the minimum extraction rate of 17%, the maximum extraction rate of 30%, and the multiplicity was set at 5 and 10 times. 200 g of tea is about the theoretical amount of tea for an adult in mice, so the low and high doses of tea extract for mice were designed to be 200 mg/kg (ZY-L) and 400 mg/kg (ZY-H), respectively, and the volume of gavage was 10 mL/kg for 28d; Cons were given water (10 mL/kg); Mods received CUMS for 4 weeks and were treated with water (10 mL/kg); Fluo received CUMS for 4 weeks and were treated with fluoxetine (2.98 mg/kg/d) (10 mL/kg) (Ministry of Health of the People’s Republic of China, 2003; Zhao and Sun, 2010; Jinhua et al., 2015).

### 2.3. Chronic unpredictable mild stress procedure

The procedure of CUMS is carried out according to the method of Wang et al. (2020), with slight modifications. In short, the CUMS process included a variety of mild stresses, including tail pinching for 2 min, slanted cage at 45 degrees for 24 h, fasting for 24 h, water deprivation for 24 h, wet Bedding for 24 h, lighting for 24 h, horizontal shaking for 6 min, swimming in ice water (4°C) for 6 min, a total of 8 stimulation methods, one stimulation method was randomly selected at 8:30 am every day, and it was not repeated within 3 days so that the animals could not foresee the stimulation given. The Con group cannot be disturbed and stimulated except for necessary procedures. Fecal sample collection was performed on the last day of the CUMS program.

### 2.4. Antidepressant-like activity

Anxiety and depression in mice were assessed using SPT, OFT, and FST, and all behavioral experiments were tested by trained observers who were blinded to the intervention.

#### 2.4.1. SPT (sugar preference test)

The sugar preference test (SPT) was used to quantify the loss of interest in rewarding stimuli. During the modeling process, sugar water experiments were carried out on 7d, 14d, 21d, and 29d, respectively. The SPT was performed as described by Yang et al. (2018), and made some modifications, which were divided into a training period and a testing period. The 2 days before the test was used as a training period to fully adapt the animals to drinking water with sucrose. The animals were given two bottles of 1% sucrose water for the first 24 h, and the mice were given one bottle of 1% sucrose water (100 mL) and one bottle of pure water for the second 24 h. After water deprivation for 24 h, the test period was entered, at the same time, the mice were given 1 bottle of 1% sucrose water and 1 bottle of pure water for 2 h. During the test, the positions of the two bottles were interchanged to avoid the effect of positional preference. At the end of the test, the consumption of sucrose solution and pure water was calculated by weighing the bottles.

Preference for sugar water (%) = consumption of sucrose water/(total amount of sucrose water + pure water) × 100%.

#### 2.4.2. OFT (open field test)

An open-field test was conducted to evaluate exploratory behavior and anxiety levels. The OFT is proceeding as described by Li et al. (2021). The apparatus for the open field test was a square wooden box (40 × 40 × 30 cm^3^) divided into 25 squares at the bottom. All mice were placed in the center of the open field apparatus and allowed to explore freely for 5 min. The number of times each mouse crossed the square (crossing the area with all four paws) and the number of times it stood (raising its front paws) were recorded. After each mouse was tested, the area was wiped with a damp cloth to avoid interference from residual odors from other mice.

#### 2.4.3. FST (forced swimming test)

The FST has been used to identify depressive-like behavior in animals. The FST was performed as previously described by Sun et al. (2018).

### 2.5. Histopathological examination

After all behavioral examinations were completed, the mice fasted for 12 h, were placed in an anesthetic chamber, and executed using isoflurane inhalation gas anesthesia. The samples were fixed with a 4% paraformaldehyde solution and Nissl staining was performed on the hippocampal tissues of mice.

Nissl staining was performed by placing mouse hippocampal paraffin sections in toluidine blue staining solution for 10 min, followed by 95% ethanol separation for a few seconds, followed by xylene transparency and neutral resin sealing, and scanning under a light microscope for subsequent image analysis. The sections were then scanned under a light microscope for image analysis. The image was opened in Image J software and processed as follows: firstly, it was converted to an 8-bit grey-scale image, then, after correction for optical density, a measurement threshold was selected for subsequent accurate quantification, and finally, the integrated optional density (IOD) was calculated for the range above the threshold. The calculated IOD reflects the total amount of nisin expression (Zhang et al., 2020).

### 2.6. Biochemical analyses

After the behavioral tests, the mice were sacrificed and the tissue of the brain was rapidly dissected for the biochemical tests. The levels of neurobiochemical factors 5-HT, DA, and BDNF as well as inflammatory factors IL-6 and TNF-α in brain tissues were determined by using mouse 5-HT, DA, BDNF, IL-6 and TNF-α ELISA kits (Cusabio, Wuhan, China).

### 2.7. 16S rRNA analysis of fecal microbiota

The experiments included extracting the total DNA from samples (n = 6 per group) of the faces. The data were analyzed on the free online Majorbio I-Sanger Cloud Platform. Total DNA was extracted according to the instructions of the E.Z.N.A.® SOIL Kit (Omega Bio-Tek, Norcross, GA, U.S.). The concentration and purity of DNA were measured using a NanoDrop 2000 spectrophotometer, and the quality of the DNA extraction was confirmed by 1% agarose gel electrophoresis. PCR amplification of the V3–V4 variable region was performed using 338F (5′-ACT CCT ACG GGA GCA GCA G-3′) and 806R (5′-GGA CTA CHVGGG TWT CTAAT-3′) primers (Liu et al., 2016). The microbial composition was analyzed via 16S rRNA sequencing by Shanghai Majorbio Bio-pharm Technology (Shanghai, China) according to standard instructions.

### 2.8. Determination of fecal metabolomics by HPLC-QTOF-MS

#### 2.8.1. Metabolite extraction

50 mg solid samples were accurately weighed, and the metabolites were extracted using a 400 μL methanol: water (4:1, v/v) solution with 0.02 mg/mL L-2-chlorophenylalanin as internal standard. The mixture was allowed to settle at −10°C and treated by High throughput tissue crusher Wonbio-96c (Shanghai wanbo biotechnology co., LTD) at 50 Hz for 6 min, then followed by ultrasound at 40 kHz for 30 min at 5°C. The samples were placed at −20°C for 30 min to precipitate proteins. After centrifugation at 13000 g at 4°C for 15 min, the supernatant were carefully transferred to sample vials for LC–MS/MS analysis.

#### 2.8.2. Metabolite extraction quality control sample

As part of the system conditioning and quality control process, aliquots of all samples are mixed to make a mixed quality control (QC) sample, and the QC sample is handled and tested in the same manner as the analyzed samples. It helps to represent the entire sample set by injecting samples at regular intervals (every 6 samples) to monitor analytical stability.

#### 2.8.3. (UHPLC–MS/MS) analysis

The instrument platform for this LC–MS analysis is the UHPLC-Q Exactive HF-X system of Thermo Fisher Scientific. Chromatographic conditions and MS conditions are referenced by Liu et al. (2023) and Shen et al. (2023).

### 2.9. Statistical analysis

Purified amplicons were pooled in equimolar amounts and paired-end sequenced on an Illumina NovaSeq PE250 platform (Illumina, San Diego, USA)^1^ according to the standard protocols by Majorbio Bio-Pharm Technology Co. Ltd. (Shanghai, China). These α diversity index (Shannon, Simpson, Chao, Sobs, Ace) were calculated for our samples using QIIME (See Footnote 1). Additionally, beta diversity was calculated using QIIME. BLAST was used for sequence alignment, and the feature sequences were annotated with the Silva database for each representative sequence. Other diagrams, such as sparse curves were obtained using the R software package (v3.5.2). The metagenomic function was predicted by PICRUSt2 (Phylogenetic Investigation of Communities by Reconstruction of Unobserved States) (Douglas et al., 2020) based on OTU representative sequences.

Spearman’s correlation coefficient analysis, one-way ANOVA, and Student’s *t*-test were performed using SPSS Statistics Version 25.0 (IBM Corp., Armonk, NY, USA). Principal component analysis (PCA), principal co-ordinates analysis (PCoA), and partial least squares discriminant analysis (PLS-DA) were performed using SIMCA 14.1 software (Umetrics, Umeå, Sweden). A heatmap was generated by MultiExperiment Viewer 4.9.0 (Oracle, Redwood, CA, USA). The Enrichment Analysis and Pathway Analysis was performed by MetaboAnalyst 4.0.^2^

## 3. Results

### 3.1. Chemical constituents of Ziyan green tea aqueous extracts

**Figure 1:**
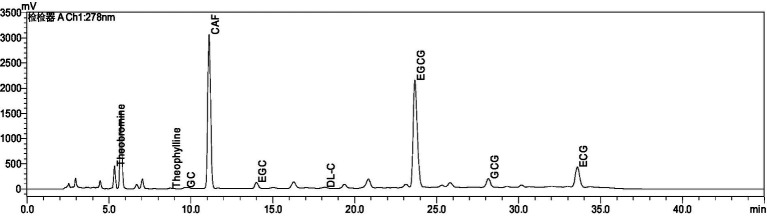
Chromatogram of the biochemical composition of aqueous extract of Ziyan green tea.

As can be seen from Supplementary Table S1 and Figure 1, we determined Amino Acid (AA), Tea Polyphenol (TPs), Soluble Sugars (SS), and Total catechins (DL-C, catechin; EC, epicatechin; EGC, epigallocatechin; ECG, epicatechin gallate; GCG, gallocatechin gallate; EGCG, epigallocatechin gallate; GC, gallocatechin), Theobromine (TB), Theophylline (THEO), Caffeine (CAF), Theaflavins (TFs), Thearubigins (TRs), Theabrownins (TBs), and Total anthocyanins (TAs). The highest content of Tea Polyphenol was 49.39%, Thearubigins was 6.77%, Total anthocyanins were 5.55%, Amino Acid was 9.90%, Caffeine was 6.68% and Soluble Sugars was 6.59% in the aqueous extract of Ziyan green tea. Total catechins 25.25%, Theaflavins 0.39%, Theabrownins 6.70%, Theobromine 0.57% and Theophylline the lowest 0.03%.

### 3.2. Ziyan green tea reduces CUMS-induced depression and improves depression-like behavior

**Figure 2:**
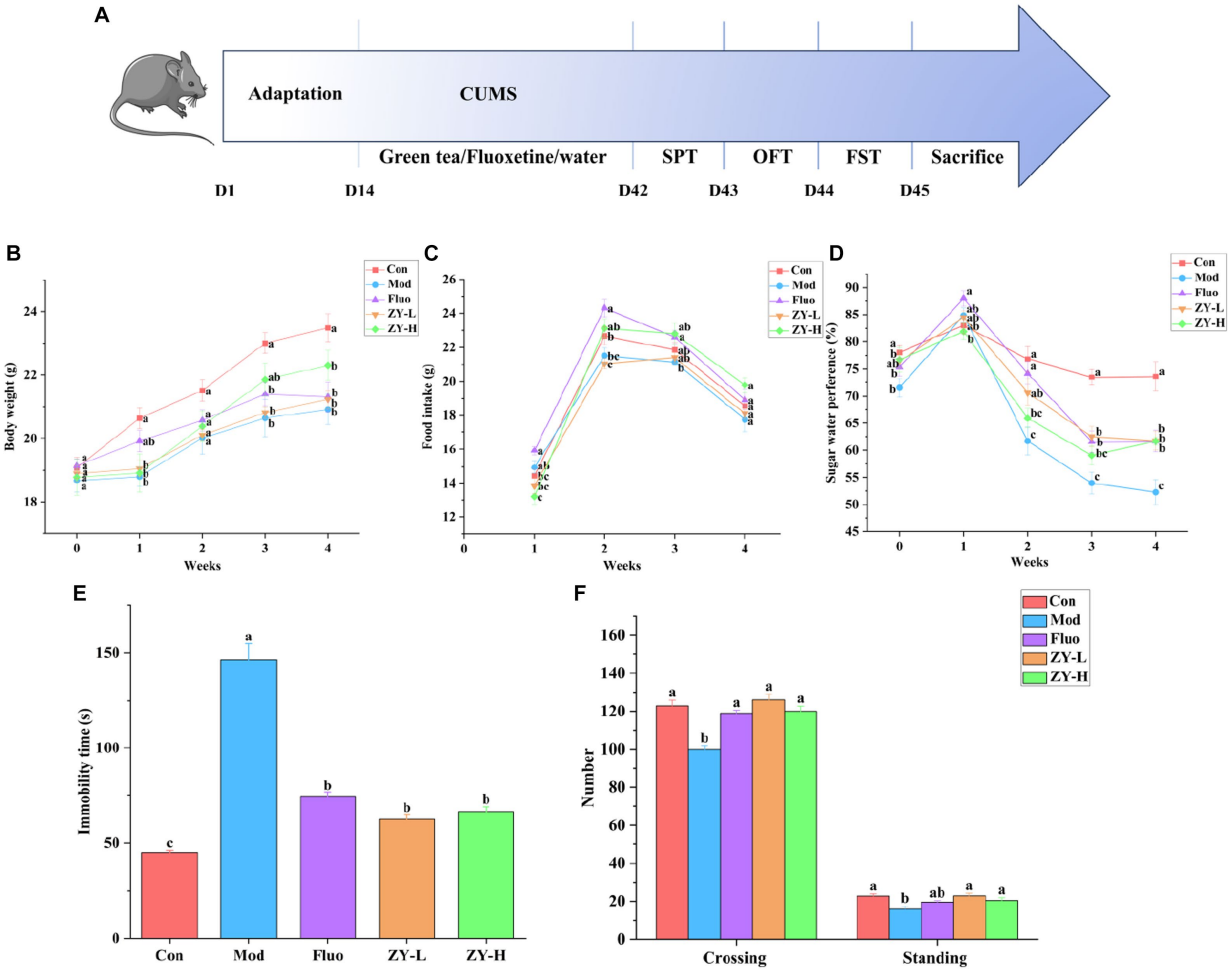
Depressive-like behavior induced by CUMS and the protective effect of Ziyan green tea. **(A)** Experimental flowchart. **(B)** Body weight. **(C)** Food intake. **(D)** Sugar water preference. **(E)** Immobility time in FST. **(F)** Number in the OFT. Con, control group; Mod, CUMS group; FLuo, CUMS mice treated with fluoxetine; ZY-L, CUMS mice treated with Ziyan green tea dhool at a low dose; ZY-H, CUMS mice treated with Ziyan green tea dhool at a high dose.

The experimental design steps are shown in Figure 2A. The CUMS induced a significant decrease in body weight as well as food intake growth levels in mice from 0–4 weeks compared to the Con. The body weight and food intake of depressed-like mice increased after ZY-L and ZY-H interventions compared to the Mod (Figures 2B,C).

As seen in Figure 2D, mice in the Mod showed significantly lower sugar-water preference compared to the Con. In contrast, the Ziyan green tea intervention group significantly increased the rate of preference for sugar water in mice (*p* < 0.05).

The results of Figure 2E showed that the resting time of mice in the Mod was significantly longer than that in the Con (*p* < 0.05); the gavage intervention with ZY-L and ZY-H significantly reduced the immobility time of CUMS mice in the forced swimming test (*p* < 0.05).

After the intervention of CUMS mice, the number of crossing and standing in the open field experiment increased significantly (*p* < 0.05), indicating that the behavioral activities and the ability to explore the unknown were restored in depressed mice, so Ziyan green tea could reduce the depression-like behavior of depressed mice in the open field experiment (Figure 2F).

### 3.3. Protective effect of Ziyan green tea on CUMS-induced hippocampal neurons in mice

**Figure 3:**
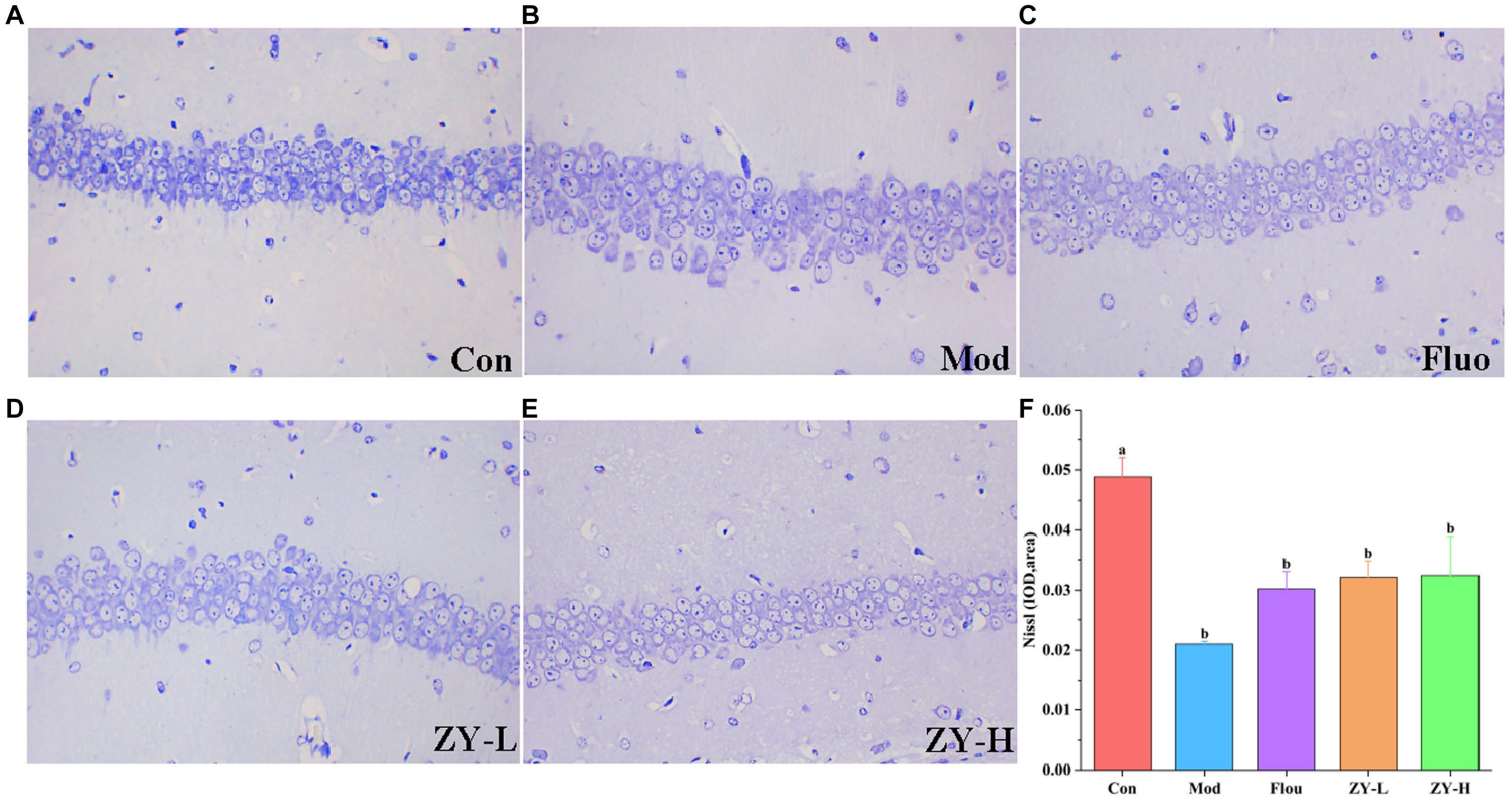
Results of Nissl staining in the hippocampus. **(A)** Con. **(B)** Mod. **(C)** Fluo. **(D)** ZY-L. **(E)** ZY-H. **(F)** IOD values of nichrome in the hippocampal region (*n* = 3).

In the Con, the hippocampal tissue was intact and clear, with neuronal cells arranged neatly and tightly, and the nuclei were obvious; the Nisin bodies was darker and more numerous. However, after CUMS modeling, the neuronal cells in mice had poor morphology, nuclei appeared solidified, fragmented, or lysed; neuronal cells were loosely arranged or had missing or even detached nuclei, and the Nisin bodies was lighter and the number was reduced. Interestingly, the degree of neuronal damage was restored after Fluo, ZY-L, and ZY-H treatment, and the number of Nisin bodies increased significantly, partially suppressing the histopathological damage (Figures 3A–F). In conclusion, Ziyan green tea inhibited neuronal damage and apoptosis in the hippocampus of CUMS-induced depressed mice to some extent and restored the number of Nisin bodies.

### 3.4. Effects of Ziyan green tea on neurochemical and pro-inflammatory factors in CUMS-induced depression

**Figure 4:**
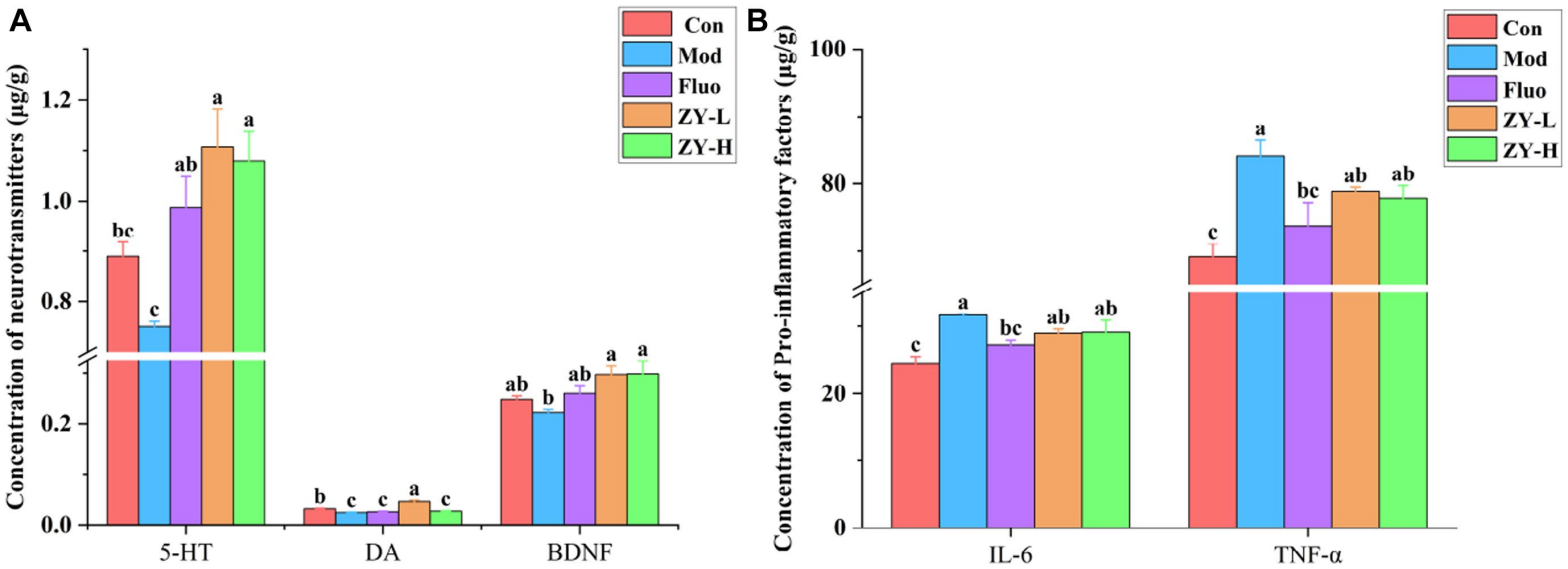
Changes in neurochemical and pro-inflammatory factors levels in the brains of depressed mice. **(A)** The level of 5-HT, DA, and BDNF in the cerebral. **(B)** The level of IL-6 and TNF-α.

5-HT neurotransmitter dysfunction or impairment has important implications in the neurobiological mechanisms of depression. The DA system plays a crucial role in various aspects of brain function, such as motor skills, emotions, and cognition, and is closely linked to reward, pleasure, and motivated behavior. Studies have shown that the levels of DA, 5-HT, and other neurotransmitters in the blood and cerebrospinal fluid of depressed animals and patients are lower compared to non-depressed individuals (Goodwin and Post, 1983; Dunlop and Nemeroff, 2007). The results of this experiment found (Figure 4A) that 5-HT and DA levels in the brain tissues of CUMS mice were significantly reduced compared with the Con; however, 5-HT levels in the brains of CUMS-induced depressed mice intervened with ZY-L and ZY-H were significantly increased (*p* < 0.05) compared with the Mod, while DA levels in the brains of ZY-L-intervened mice were significantly increased (*p* < 0.05). BDNF levels were reduced in the brains of depressed animals and patients, and antidepressants restored BDNF levels and reversed their behavioral and cellular effects (Rothenberg and Zhang, 2019). In the present study, ZY-L and ZY-H significantly increased intracerebral BDNF levels compared to the Mod (*p* < 0.05) (Figure 4A).

Elevated IL-6 may lead to HPA axis dysfunction, altered synaptic neurotransmission, and reduced neurotrophic factors, which are indirectly involved in the pathogenesis of depression (Nukina et al., 2001). Postal and Appenzeller (2015) confirmed by Meta-analysis that TNF-α was significantly elevated in patients with chronic insomnia with anxiety and depression and suggested that reducing serum TNF-α levels could improve patients’ depressive symptoms. In this study, we found that IL-6 and TNF-α were significantly elevated in the brains of CUMS mice compared with Con (*p* < 0.05), and it was known from Figure 4B that the levels of IL-6 and TNF-α in the brains of CUMS mice were reduced to some extent after ZY-L and ZY-H interventions compared with the Mod.

The results showed that Ziyan green tea could improve the depressive symptoms induced by CUMS by increasing the expression of 5-HT, DA, and BDNF in the brain of CUMS-induced mice, and reducing the levels of pro-inflammatory factors IL-6 and TNF-α.

### 3.5. Ziyan green tea modulates the microbial composition of CUMS mice

Previous research has shown that changes in the gut microbiome affect depression-like behaviors (Lucidi et al., 2021). Therefore, we attempted to determine whether CUMS-exposed mice exhibited alterations in the gut microbiome. It can be seen in Table 1 that the Coverage is >0.99, which proves that the sequencing data in this study reached saturation and the sequencing depth can cover most of the species in the mouse gut microbiome community, which is sufficient to reflect the diversity contained in the given samples. Alpha diversity was analyzed by calculating the Shannon, Simpson, Chao, Ace, and Sobs indices on the OTU level. The results show that compared with the Con, the diversity and richness of the intestinal microbiota were significantly reduced after external stimulation, and the results of Coverage, Shannon, and Simpson indices indicated that the diversity of intestinal microbiota was reduced in the Mod, and the Sobs, Ace, and Chao indices indicated that the richness of intestinal microbiota was significantly lower in the Mod than in the Con. After treatment with Ziyan green tea, the microbial community diversity and richness of CUMS mice was significantly restored.

**Table 1:** Comparison of microbial diversity indices and coverage of 16S rRNA gene libraries at 97% similarity based on the sequencing analysis.

Processing stage	Coverage	Shannon	Simpson	Chao	Sobs	Ace
Con	0.9920 ± 0.0020 a	3.4794 ± 0.4118 a	0.0747 ± 0.0177 b	729.1074 ± 76.191 a	554.3333 ± 80.5647 a	859.9592 ± 50.769 a
Mod	0.9900 ± 0.0024 a	3.1259 ± 0.4401 a	0.1368 ± 0.0357 a	703.0145 ± 75.8334 a	509.0000 ± 55.2702 a	709.1814 ± 77.3436 b
Fluo	0.9903 ± 0.0015 a	3.4745 ± 0.4955 a	0.1187 ± 0.0468 ab	799.5941 ± 43.7799 a	562.3333 ± 84.4693 a	903.0616 ± 80.6407 a
ZY-L	0.9904 ± 0.0010 a	3.3485 ± 0.4168 a	0.1166 ± 0.0254 ab	792.5531 ± 65.769 a	550.6667 ± 62.1761 a	872.8476 ± 110.465 a
ZY-H	0.9905 ± 0.0013 a	3.5385 ± 0.2205 a	0.1060 ± 0.0138 ab	778.1627 ± 64.2526 a	554.5000 ± 68.3308 a	887.6237 ± 143.6439 a

**Figure 5:**
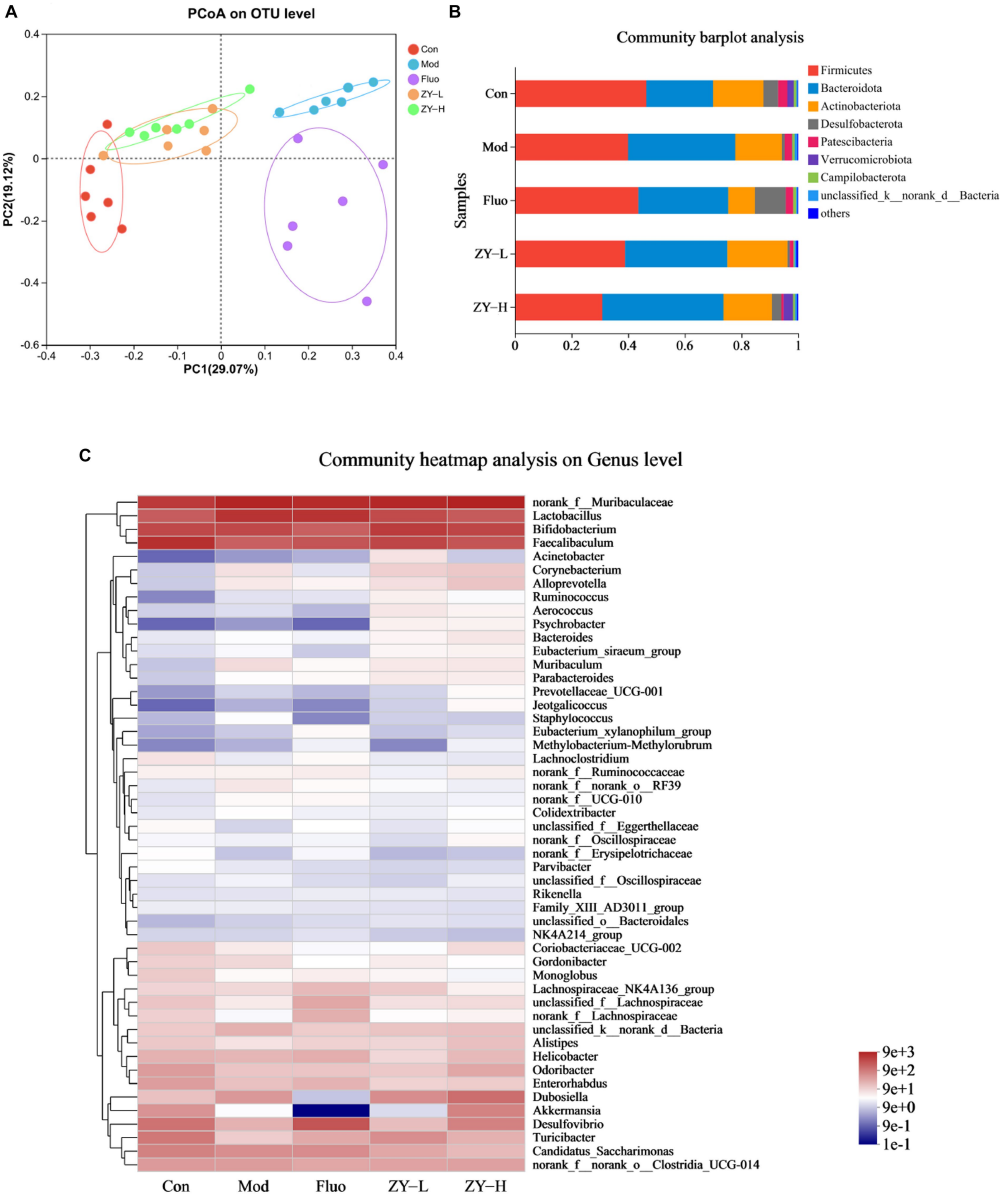

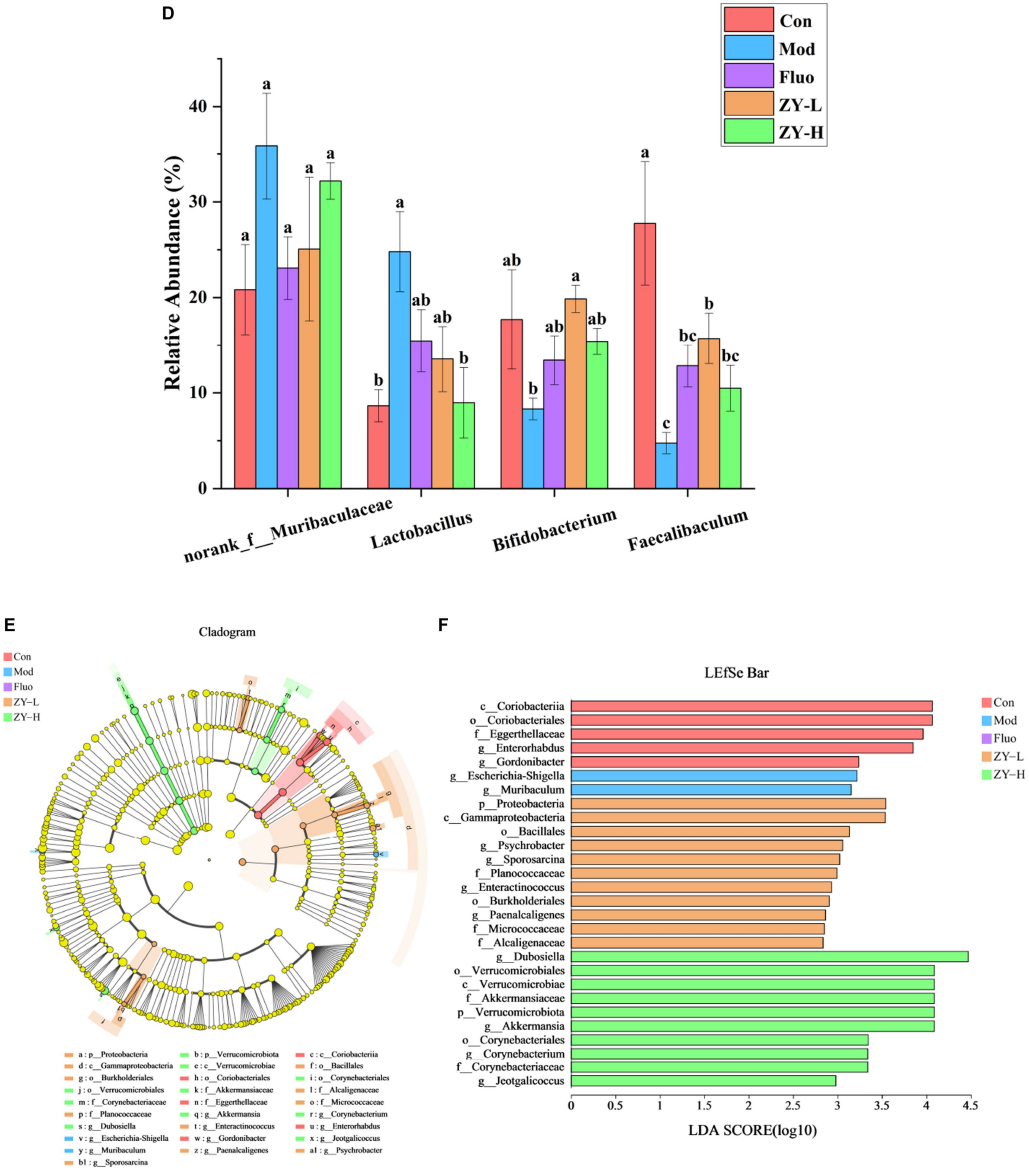
Effects of Ziyan green tea on the microbial composition of CUMS mice. **(A)** Principal coordinate analysis. **(B)** Community barplot analysis on phylum level. **(C)** Community heatmap analysis on Genus level. **(D)** Relative abundance of distinguishable genu. **(E)** Cladogram analysis among different groups. **(F)** Column chart of linear discriminant analysis (LDA).

Beta diversity was analyzed by PCoA plots, and differences in microbial composition (OTU) among the five groups were assessed using the nonphylogenetic Bray-Curtis metric (Figure 5A). The distance between different samples was calculated using the variation in abundance between samples, it could be seen that the microbiota of the five groups was not completely clustered together, on the first principal component (PC1) axis, there is a clear separation between the Mod and Con, and a between-group difference test was performed based on ANOSIM, *p* = 0.001, a highly significant difference between groups, suggesting that CUMS-induced depression can alter the microbiota structure.

To further explore the detailed composition of the intestinal microbiota of the five groups of mice, the relative abundance was analyzed at two taxonomic levels of phylum and genus. At the phylum level, Firmicutes, Bacteroidetes, Actinobacteriota, and Desulfobacterot were the most abundant phylum among all groups, accounting for more than 90% of the total bacterial community (Figure 5B).

At the genus level, the variation in abundance of four bacterial genera, *norank_f_Muribaculaceae*, *Lactobacillus*, *Bifidobacterium*, and *Faecalibaculum*, was analyzed (Figures 5C,D), and the relative abundance of *norank_f_Muribaculaceae*, *Lactobacillus* in Mod was increased compared to Con. The abundance level of the *norank_f_Muribaculaceae* genus decreased by 35.59% after fluoxetine administration, and the abundance decreased by 30.02% after ZY-L intervention. As for the genus *Lactobacillus*, it is worth mentioning that the genus abundance level significantly decreased by 63.86% after ZY-H intervention (*p* < 0.05), and 37.62 and 45.37% for *Lactobacillus* after Fluo and ZY-L interventions, respectively. In addition, the relative abundance of *Bifidobacterium* and *Faecalibaculum* in Mod decreased compared to Con and significantly increased by 58.16 and 69.79% after ZY-L intervention, respectively (*p* < 0.05), and the relative abundance of *Bifidobacterium* and *Faecalibaculum* was also increased after the other Fluo and ZY-H treatments, which could reverse this change.

To explore the specific bacterial taxa associated with CUMS-induced depression and Ziyan green tea intervention, a LEfSe evolutionary branching map was presented between treatments. Firstly, the nonparametric factorial Kruskal-Wallis rank sum test was used to test the characteristics with significant differences in abundance among different samples to determine the groups with significant differences in abundance (LDA > 2). Branching plots showed (Figure 5E) that Verrucomicrobia, Bacteroidetes, Firmicutes, Actinobacteriota, and Proteobacteria at the phylum level were enriched significantly among each group. LEfSe analysis was performed to identify specific bacterial taxa that could distinguish between samples to account for differences in taxa from phylum to genus level on the phylogenetic tree of bacterial communities in the samples. Among them, 28 bacterial branches showed statistically significant differences (Figure 5F), and there were 5 genera with different taxonomic levels in the Con, including c__Coriobacteriia, o__Coriobacteriales, f__Eggerthellaceae, *Enterorhabdus*, *Gordonibacter*; however, only two distinguishable bacterial taxa were detected in the Mod, *Escherichia-Shigella* and *g__Muribaculum*. The enrichment and abundance of the bacteria were significantly increased after intervention with Ziyan green tea, there was 11 enrichment flora in the ZY-L, p__Proteobacteria, c__Gammaproteobacteria, o__Bacillales, *Psychrobacter*, *Sporosarcina*, f__Planococcaceae, *Enteractinococcus*, o__Burkholderiales, *Paenalcaligenes*, f__Micrococcaceae, and f__Alcaligenaceae. With 10 genera in the ZY-H, mainly including *Dubosiella*, o__Verrucomicrobiales, c__Verrucomicrobiae, f__Akkermansiaceae, p__Verrucomicrobiota, *Akkermansia*, o__Corynebacteriales, *Corynebacterium*, f__Corynebacteriaceae and *Jeotgalicoccus*. LEfSe Bar analysis further showed that the enrichment of intestinal flora in mice was reduced after external stimulation and different doses of Ziyan green tea soup intervened to change the enrichment of intestinal flora thus reducing the depressive symptoms.

We used PICRUSt2 functional prediction to predict the functional information of the microbial community in our samples to further our understanding of some potential microbial functional features during disease development through functional composition and abundance. And based on 16S rRNA sequencing data, we analyzed all the samples between different Kyoto Encyclopedia of Genes and Genomes (KEGG) tertiary groups. The results showed that among all KEGG pathways, the abundance of metabolic pathways and biosynthesis of secondary metabolites were the most correlated (Supplementary Figure S1).

### 3.6. Analysis of metabolite differences among groups after intervention with Ziyan green tea

#### 3.6.1. Identification of differential metabolites in feces of CUMS mice

**Figure 6:**
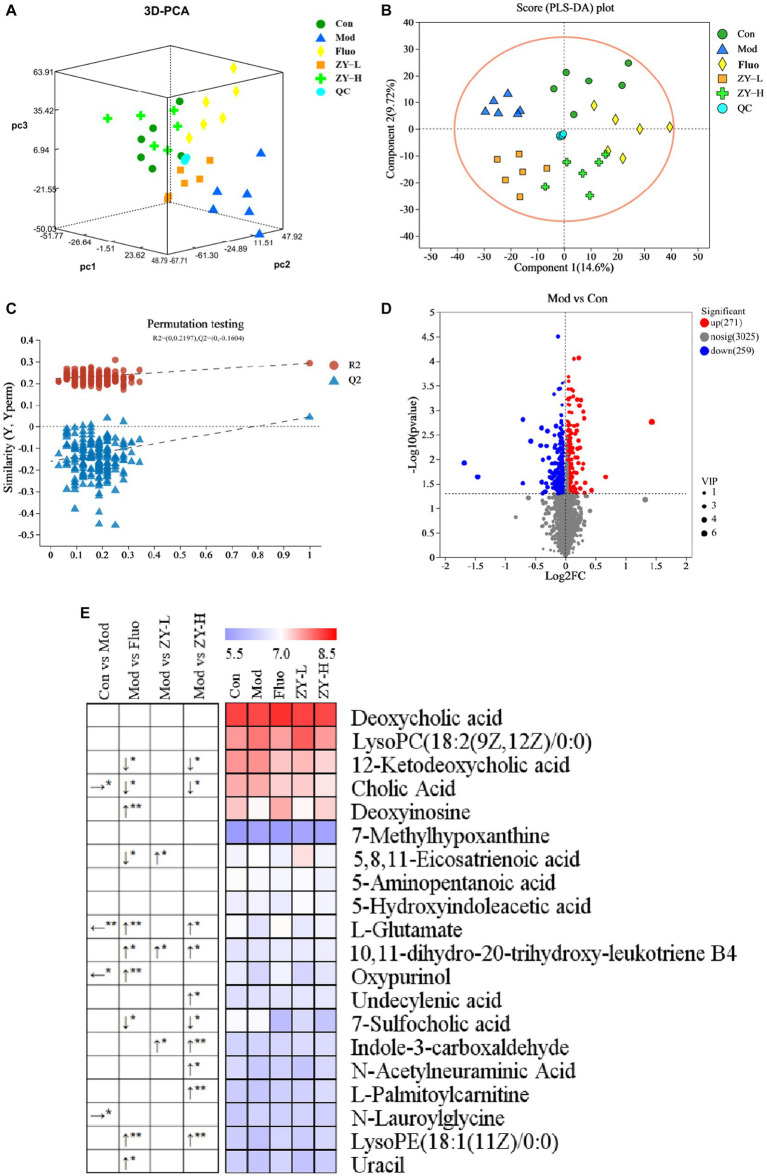
**(A)** 3D PCA score chart. **(B)** PLS-DA among five groups. **(C)** PLS-DA corresponding of 200 random permutation test plots. **(D)** Volcano map for differential metabolite screening (Mod vs. Con). **(E)** Heat map of differential metabolite content in different treatment groups. The left arrow (←) and right arrow (→) represent that the relative content of differential metabolites was significantly down-regulated and up-regulated after the CUMS intervention compared with the Con. The arrows (↑) and (↓) represent that the relative content of differential metabolites was significantly up-regulated and down-regulated after the intervention of different doses of Ziyan green tea compared with the Mod. *p < 0.05, **p < 0.01.

PCA is an unsupervised multivariate statistical method, which can reveal the internal structure of the overall sample and can visually describe the change trends of different groups by the trajectory of each group on the principal component coordinate graph. PCA clustering analysis was performed on the fecal samples of each group to obtain the trends of fecal metabolism changes shown in Figure 6A, and the results showed that there were significant differences in the distribution of metabolic profiles among the five groups. In order to observe the CUMS-induced fecal metabolic differences in depth, the experiment hoped to model and analyze the samples of each group by PLS-DA to find the metabolic changes associated with CUMS-induced depressive behavior, which can be seen in Figure 6B, the Mod and Con clustered into two distinct independent parts, and there was no crossover in the distribution between their samples, indicating that there were significant metabolic differences between the Mod and Con, the modeling was successful, suggesting that CUMS stimulation changed the fecal metabolic profile of the mice. The model validation results showed that all R^2^ and Q^2^ values on the left side were lower than the original points on the right side, and the regression line of Q^2^ intersected with the vertical axis with values less than 0 (R^2^Y = 0.2197, Q^2^ = −0.1604), indicating that the constructed model had high reliability (Figure 6C). Meanwhile, focusing on each dosing group, it can be found that the clustering areas of ZY-L, ZY-H, and Fluo can be distinguished from the Mod, indicating that the aqueous extract of Ziyan green tea also has a therapeutic effect on depression, which is consistent with the results in the previous behavioral evaluation experiments. The clustering area of the Con was relatively farther from the Mod than that of the Ziyan green tea group, suggesting that Ziyan green tea has a lower effect on the recovery of the metabolic network than the positive control drug fluoxetine.

To further identify the differential metabolites among the groups and to clarify the modulating effects of ZY-L, ZY-H, and Fluo on fecal differential metabolites, a total of 20 metabolites with significantly different peak areas were obtained by Volcano map (Figure 6D; Supplementary Figure S2) combined with VIP values (>1) and One-way analysis of variance (*p* < 0.05): Deoxycholic acid, LysoPC(18:2(9Z,12Z)/0:0), 12-Ketodeoxycholic acid, Cholic Acid, Deoxyinosine, 7-Methylhypoxanthine, 5,8,11- Eicosatrienoic acid, 5-Aminopentanoic acid, 5-Hydroxyindoleacetic acid, L-Glutamate, 10,11-dihydro-20-trihydroxy-leukotriene B4, Oxypurinol, Undecylenic acid, 7-Sulfocholic acid, Indole-3-carboxaldehyde, N-Acetylneuraminic Acid, L-Palmitoylcarnitine, N-Lauroylglycine, LysoPE(18. 1(11Z)/0:0) and Uracil (Figure 6E). Cholic Acid and N-Lauroylglycine levels were significantly higher, while L-Glutamate and Oxypurinol were significantly lower in the fecal samples of the Mod when compared to the Con (*p* < 0.05). After Fluo intervention, 12-Ketodeoxycholic acid, Cholic acid, 5,8,11-Eicosatrienoic acid, and 7-Sulfocholic acid were significantly lower compared to the Mod, while Deoxyinosine, L-Glutamate, 10,11-dihydro-20-trihydroxy-leukotriene B4, Oxypurinol, Undecylenic acid, LysoPE(18:1(11Z)/0:0) and Uracil were significantly higher. The relative levels of 5,8,11-Eicosatrienoic acid, 10,11-dihydro-20-trihydroxy-leukotriene B4 and Indole-3-carboxaldehyde were significantly higher after ZY-L intervention. After ZY-H intervention, the contents of 12-Ketodeoxycholic acid, Cholic Acid, and 7-Sulfocholic acid were significantly lower compared to the Mod, while the relative contents of L-Glutamate, 10,11-dihydroxy-20-trihydroxy-leukotriene B4 and Indole-3-carboxaldehyde were significantly higher. 10,11-dihydro-20-trihydroxy-leukotriene B4, Undecylenic acid, Indole-3-carboxaldehyde, N-Acetylneuraminic Acid, L-Palmitoylcarnitine and LysoPE (18:1(11Z)/0:0) were significantly elevated.

#### 3.6.2. Metabolic pathway analysis

**Figure 7:**
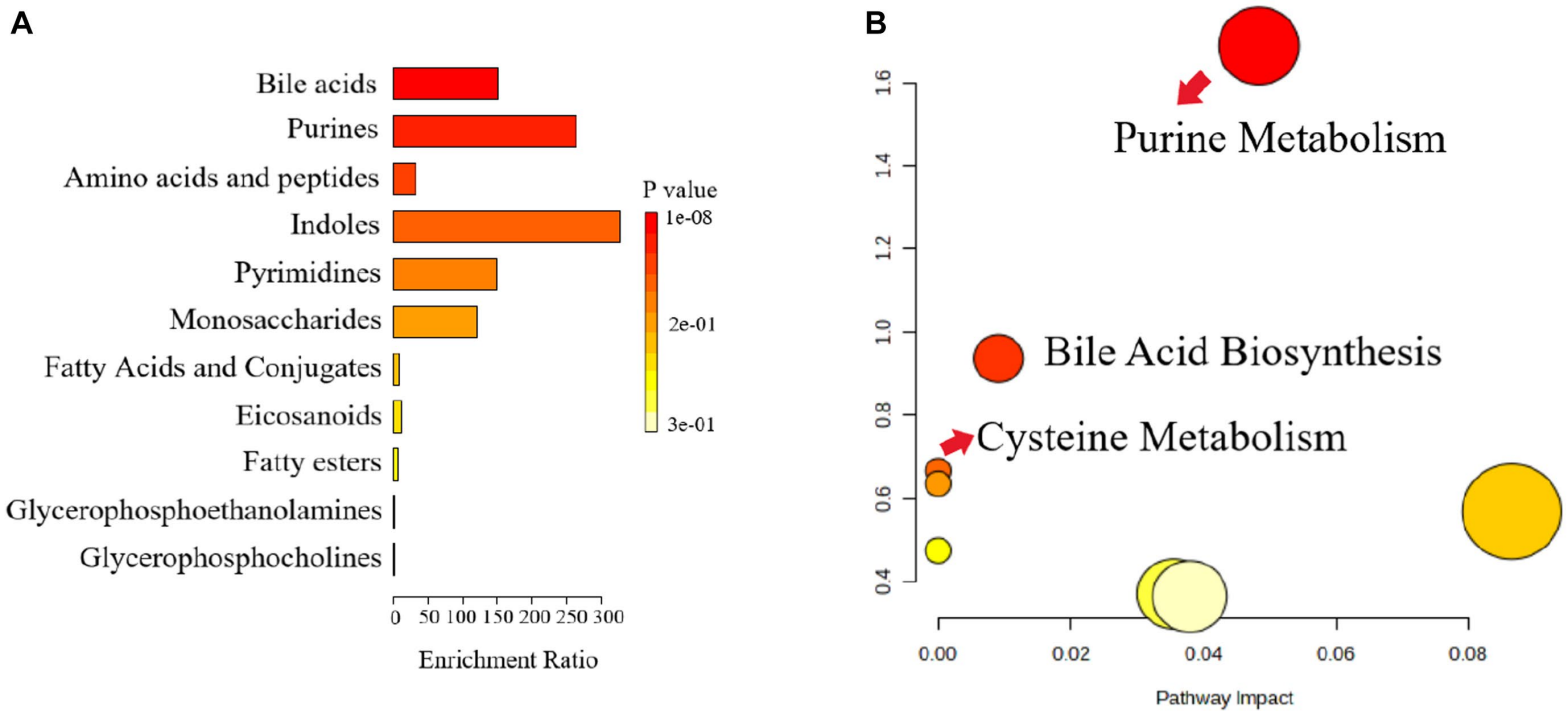
Metabolic pathway enrichment analysis. **(A)** Histogram, the darker the colour, the smaller the value of *p*, and the more pronounced the enrichment of the corresponding metabolic pathway. **(B)** Bubble chart, Each bubble represents a metabolic pathway, and the horizontal coordinates of the bubble and the size of the bubble indicate the influence factor of the pathway in the enrichment analysis, the larger the bubble, the larger the influence factor; the vertical coordinates of the bubble and the color of the bubble indicate the value of *p* of the enrichment analysis (taking the negative common logarithm, i.e., -log_10_p), the darker the color, the smaller the value of *p*, the more significant the enrichment.

The pathway analysis can provide biological information on the relevant metabolites, which can help us to further understand the pathogenesis of depression and the antidepressant effect of Ziyan green tea. Based on the identified potential differential metabolites, the relevant metabolic pathways were identified by applying databases such as KEGG^3^ and HMDB^4^ as well as enrichment analysis. The results of the enrichment analysis (Figure 7A) showed that Bile Acid was the most relevant in this analysis, and the metabolic pathway analysis identified three metabolic pathways that were most relevant to the depression-like behavior of CUMS mice (Figure 7B; Supplementary Table S1), including Purine Metabolism, Bile Acid Biosynthesis and Cysteine Metabolism. These results suggest that Ziyan green tea mainly affects Purine Metabolism, Bile Acid Biosynthesis, and Cysteine Metabolism in the intestinal tract of CUMS mice.

### 3.7. Correlation between intestinal flora and metabolites, neurobiochemical factors, and pro-inflammatory factors in mice after intake of Ziyan green tea

The relationship between gut microbes and host metabolism is gaining attention, and to investigate whether gut microbes may mediate the effects of antidepressants on the organism by participating in host metabolism, we further performed Spearman’s rank correlations Heat Map for five groups, selecting the top 20 microbes in terms of genus-level colony abundance with the aforementioned differential metabolites, neurobiochemical and pro-inflammatory factors, to assess the potential contribution to alleviating CUMS-induced depression. The results showed that a total of four microbial communities at the genus level showed significant correlations with neurobiochemical factors, six genera were significantly correlated with inflammatory factors, and 13 genera were significantly correlated with fecal differential metabolites.

**Figure 8:**
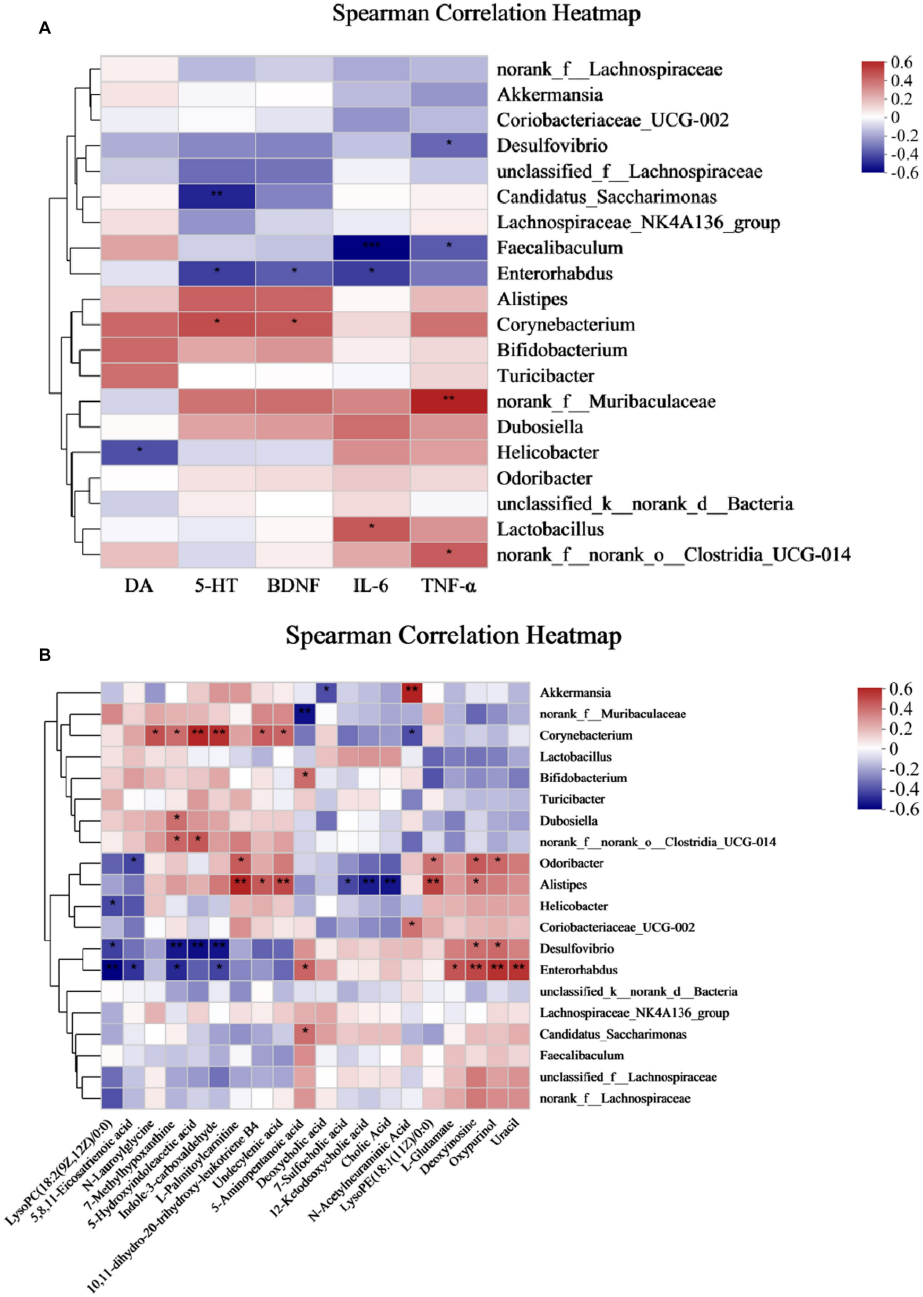
Spearman correlation Analysis Heat Map. **(A)** Spearman’s rank correlation between the intestinal flora and neurobiochemical and pro-inflammatory factors in CUMS mice. **(B)** Spearman’s rank correlation between the intestinal flora and differential metabolites. (*p < 0.05, **p < 0.01, ***p < 0.001).

From Figure 8A, *Corynebacterium* showed a significant positive correlation with 5-HT and BDNF, *Faecalibaculum* showed a significant negative correlation with IL-6 and TNF-α, *Enterorhabdus* showed a significant negative correlation with IL-6, and *Desulfovibrio* showed a significant negative correlation with TNF-α. Therefore, *Corynebacterium*, *Faecalibaculum*, *Enterorhabdus*, and *Desulfovibrio* had a positive intervention effect on CUMS-induced depression. Interestingly, these genera, which were significantly positively correlated with neurobiochemical factors and negatively correlated with pro-inflammatory factors, were significantly increased after Ziyan green tea. In conclusion, these findings reveal that the gut microbiota plays a key role in modulating CUMS-induced depression after Ziyan green tea intervention. A causal relationship may exist between the gut microbiota and the relative abundance of neurobiochemical factors (5-HT, DA, and BDNF in brain tissue) and pro-inflammatory factor indicators (IL-6 and TNF-α).

As seen from Figure 8B, some intestinal bacteria were significantly associated with only one metabolite, e.g., *norank_f__Muribaculaceae* was significantly negatively associated with 5-Aminopentanoic acid, *Bifidobacterium* was significantly positively associated with 5-Aminopentanoic acid; while some were associated with multiple metabolites, such as *Corynebacterium* with N-Lauroylglycine, 7-Methylhypoxanthine, 5-Hydroxyindoleacetic acid, Indole-3-carboxaldehyde, Undecylenic acid, 10,11-dihydro-20-trihydroxy-leukotriene B4 showed significant positive correlation, on the contrary, N-Acetylneuraminic Acid showed a significant negative correlation. Here we focused on two aspects: first, the metabolites produced by the direct or indirect metabolism of the intestinal flora, and second, a particular intestinal bacterium that correlates with multiple metabolites, which is important to explore the involvement of the intestinal flora in the pathogenesis of the host. It was found that *Akkermansia* was significantly positively correlated with N-Acetylneuraminic Acid and negatively correlated with Deoxycholic acid; *Alistipes* was significantly positively correlated with L-Palmitoylcarnitine, 10,11- dihydro-20-trihydroxy-leukotriene B4, Undecylenic acid, LysoPE(18:1(11Z)/0:0) and Deoxyinosine, however, 7-Sulfocholic acid, 12-Ketodeoxycholic acid, and Cholic Acid were significantly negatively correlated with it; *Desulfovibrio* was significantly positively correlated with Deoxyinosine and Oxypurinol, LysoPC(18:2(9Z,12Z)/0:0), 7-Methylhypoxanthine, 5-Hydroxyindoleacetic acid, Indole-3-carboxaldehyde showed significant negative correlations with it; *Enterorhabdus* was significantly negatively correlated with LysoPC(18:2(9Z,12Z)/0:0), 5,8,11-Eicosatrienoic acid, 7-Methylhypoxanthine, and Indole-3-carboxaldehyde, while 5-Aminopentanoic acid, L-Glutamate, Deoxyinosine, Oxypurinol, and Uracil were significantly positively correlation.

In addition, *Corynebacterium* was significantly positively correlated with 5-HT, BDNF, and *Corynebacterium* showed significant positive correlations with N-Lauroylglycine, 7-Methylhypoxanthine, 5-Hydroxyindoleacetic acid, Indole −3-carboxaldehyde, Undecylenic acid, and 10,11-dihydro-20-trihydroxy-leukotriene B4. It suggests that these metabolites may be inextricably linked to the stimulation, synthesis, or increase of neurotransmitters in the brain of depressed mice. *Enterorhabdus* and *Desulfovibrio* were significantly negatively correlated with IL-6, TNF-α, and *Desulfovibrio* and *Enterorhabdus* were significantly correlated with the following metabolites LysoPC (18:2(9Z,12Z)/0:0), 5,8,11-Eicosatrienoic acid, 7-Methylhypoxanthine, 5-Hydroxyindoleacetic acid, and Indole-3-carboxaldehyde, suggesting that such metabolites could potentially play an important role in reducing the levels of pro-inflammatory factors.

## 4. Discussion

Depression is one of the most common mental disorders in the world. The core symptom of depression is a lack of pleasure, which can be reflected by SPT and food intake (Qiao et al., 2020). CUMS has been widely used to induce an animal model of depression that mimics several human depressive symptoms as well as key biochemical signs of depression (Sharma and Thakur, 2015). Our study showed that the body weight, food intake, and sugar-water preference of depressed-like mice were significantly higher after Ziyan green tea intervention than in the Mod, improving the well-being of CUMS-induced depressed mice. After the gavage intervention of ZY-L and ZY-H, the immobility time of CUMS mice was significantly shortened in the compulsive swimming experiment, moreover, the number of crossing and standing in the open field experiment were significantly increased, indicating that the behavioral activities and the ability to explore the unknown were restored in the depression-like mice, and Ziyan green tea could improve the depression-like behavior of CUMS mice.

5-HT plays an important role in modulating mood, emotion, and behavior in stress response (Mayer et al., 2015). People with depression have lower levels of 5-HT and DA compared to healthy individuals (Hu, 2017). The level of BDNF in the brain of depressed animals and patients decreases, and antidepressant drugs can restore the level of BDNF and reverse the devaluation effect of its behavior and cells (Takebayashi et al., 2012). Our study showed that Ziyan green tea significantly modulated the levels of 5-HT, DA, and BDNF in the brain compared to Mod. Nukina et al. (2001) tested whether stress leads to elevated plasma IL-6 in mice and found that plasma IL-6 was elevated in mice after 1 h of stress and that elevated IL-6 may lead to HPA axis dysfunction, altered synaptic neurotransmission, and reduced neurotrophic factors, which are indirectly involved in the pathogenesis of depression. It has been shown that depressive symptoms in depressed patients can be improved by lowering serum TNF-α levels, which in turn improves sleep (Postal and Appenzeller, 2015). In the present study, we found that the brain levels of IL-6 and TNF-α in CUMS mice were significantly reduced and restored to normal levels after Ziyan green tea intervention, indicating that Ziyan green tea could indirectly interfere with the development of depression by reducing the levels of pro-inflammatory factors in CUMS mice.

Green tea polyphenols (GPTs) can promote energy conversion in mammals by modulating gut microbial community structure, gene homologs, and metabolic pathways (Zhou et al., 2020). Dietary supplements with tea had positive effects on maintaining intestinal microecology (Liu et al., 2019). The results of this study showed that the degree of neuronal damage was restored after ZY-L and ZY-H treatment, and the number of Nisin bodies was significantly increased, partially inhibiting histopathological damage. Therefore, Ziyan green tea can inhibit neuronal damage and apoptosis in the hippocampus of CUMS-induced depressed mice to some extent and restore the number of Nisin bodies.

There is growing evidence that dysbiosis of gut microbiota has been associated with a variety of neuropsychiatric disorders including major depressive disorder (MDD) (Chang et al., 2022). In this study, we found that CUMS induced significant changes in intestinal flora by PCoA analysis, while low and high doses of Ziyan green tea reversed the CUMS-induced changes in intestinal flora to some extent, indicating its intervention effect on intestinal flora of CUMS mice. The results of Coverage, Shannon and Simpson indices indicated that the intestinal microbiota diversity was reduced in CUMS-induced mice, and the Sobs, Ace and Chao indices indicated that the intestinal microbiota richness was significantly lower in CUMS-induced mice than in healthy mice. The microbial community diversity and richness of CUMS mice were significantly restored after Ziyan green tea treatment. Studies have shown that in the gut microbiota of alcohol-treated mice, the abundance of the harmful bacteria *norank_f_Muribaculaceae* increased and the abundance of the beneficial bacteria *Akkermansia* decreased (Yang et al., 2020; Li et al., 2021). Previous studies have shown that *norank_f_Muribaculaceae* is strongly associated with HPA axis function, correlates with 5-HT and BDNF in the hippocampus, and that the abundance of *norank_f_Muribaculaceae* was significantly higher after TG (Total glycosides) treatment compared to CUMS (*p* < 0.05) (Fan et al., 2021). Studies have demonstrated that HA (heat acclimation) alleviates depression by remodeling the gut flora f_Muribaculaceae and *g_Lactobacillus* levels were significantly decreased in the EMF (electromagnetic field) group, and HA reversed the equilibrium of gut microbes induced by EMF and significantly increase the proportion of probiotics (g_Lactobacillus) (*p* < 0.05) (Luo et al., 2021). Jasmine tea has been shown to modulate depressive symptoms by downregulating *Lactobacillus* (Zhang et al., 2022). Preparation of 100 mg of mixed tea catechins (approximately a cup of green tea) three times daily for three weeks induced a significant increase in *Lactobacillus* species in broiler chickens (Hara, 1997). More recently, 4% green tea powder supplementation for 22 weeks in HFD mice significantly increased *Lactobacillus* species, in both number and diversity (Axling et al., 2012). Some studies have shown that psychosocial and psychophysical stress alters the intestinal flora and leads to a decrease in the number of *Lactobacillus* and *Bifidobacterium*, an important factor in depression (Gulbins et al., 2018; Zhang et al., 2021). *Bifidobacteria* and *Lactobacillus* in the gut have been suggested to have beneficial effects on stress response and depression. Existing studies have demonstrated a reduction in the number of these bacteria in patients with major depressive disorder (MDD) compared to healthy controls (Aizawa et al., 2016; Liu et al., 2016). Fu instant tea improved intestinal microbiota composition reduced the ratio of F/B, and increased the abundance of beneficial bacteria, including *Lactobacillus* and *Bifidobacteria* (Yang et al., 2021). EGCG, GCG, and EGCG3′′Me, those tea polyphenols exhibited proliferative effects on the growth of *Bifidobacterium and Lactobacillus/Enterococcus groups* (Zhang et al., 2013). In our study, the Mod group *norank_f_Muribaculacea*, *Lactobacillus* was elevated, and the abundance of these two genera decreased significantly after the intervention of Ziyan green tea. *Bifidobacterium* has been associated with beneficial psychobiological effects (Rothenberg and Zhang, 2019), *Bifidobacterium breve CCFM1025* was validated to have an antidepressant-like effect in mice, supplementation of *Bifidobacterium breve CCFM1025* and *Bifidobacterium breve Bif11* to depressed patients and animals can alleviate their depressive behaviors, and their antidepressant mechanisms include attenuating over-responsiveness of the HPA axis and inhibiting the expression of inflammatory factors, enhancing the expression of 5-hydroxytryptophan (5-HT) in the intestines and the brain, and ameliorating the damage of intestinal permeability (Tian et al., 2020, 2022; Sushma et al., 2023). *Faecalibaculum* was associated with NCPB (negative cognitive processing bias) and depressive symptoms, it was demonstrated that NCPB levels were positively correlated with depressive symptoms and anxiety symptoms (*p* < 0.01). There was a significant difference in the β-diversity of microbiota in young adults between high and low NCPB groups. *Faecalibaculum* abundance was shown to be significantly higher in the High-status NCPB treatment group (Xu H. et al., 2023). It agrees with our findings; our study showed that the relative abundance of *Bifidobacterium* and *Faecalibaculum* significantly increased after Ziyan green tea intervention. The GM (gut-brain axis) plays an important role in initiating signal transduction and communication between the gut and the central nervous system (Chen et al., 2017). It has been found that jasmine tea has a significant restorative effect on microorganisms and has a significant relationship with neurotransmitters. These genera, *unclassified_f__Lachnospiraceae*, *norank_f__Desulfovibrionaceae*, et al., had a positive relationship with 5-HT and BDNF in the hippocampus and cerebral cortex (Zhang et al., 2022). Our study found that *Corynebacterium* showed a significant positive correlation with 5-HT and BDNF, *Faecalibaculum* showed a significant negative correlation with IL-6 and TNF-α, *Enterorhabdus* showed a significant negative correlation with IL-6, and *Desulfovibrio* showed a significant negative correlation with TNF-α. Experiments have shown that *Corynebacterium* abundance was significantly reduced in chronic variable stress (CVS)-induced depression rats compared to normal controls (Yu et al., 2017). Current research indicates that CUMS stimulation reduced *Enterorhabdus* abundance, which was reversed by venlafaxine treatment. So *Enterorhabdus* are the key bacteria responsible for venlafaxine-ameliorated depression in mice (Shen et al., 2023). In a rat model of ACTH-induced depression, chlorogenic acid exerted anti-depressive effects by increasing the relative abundance of *Bifidobacterium* and reducing the relative abundance of *Desulfovibrio* (Song et al., 2019). Therefore, *Corynebacterium*, *Faecalibaculum*, *Enterorhabdus*, and *Desulfovibrio* have a positive intervention effect on depression caused by CUMS.

There is much evidence that gut microbiota composition is closely related to host metabolism (Koh and Bäckhed, 2020). The results of our study showed that Bile Acid was the most enriched, and the metabolic pathway analysis identified three metabolic pathways that were most associated with depression-like behavior in CUMS mice, including Purine Metabolism, Bile Acid Biosynthesis, and Cysteine Metabolism. The size and composition of the bile acid pool appear to be important factors regulating the structure of the human gut microbial community (Ridlon et al., 2014). Bile acids have direct antimicrobial effects on gut microbes, and clinical studies have shown that bile acids are important components of the gut-brain axis, suppress neuroinflammation, and mediate the pathophysiology of Major depressive disorder (MDD) (Begley et al., 2005; Bao et al., 2021). It has been found that serum bile acid concentration was significantly increased in model rats (Xiong et al., 2016). *Alistipes* have been shown to be pathogenic in colorectal cancer and associated with psychiatric symptoms of depression (Parker et al., 2020). This is consistent with our findings, where we found that *Alistipes* were significantly and negatively correlated with 7-Sulfocholic acid, 12-Ketodeoxycholic acid, and Cholic acid. Depression leads to a significant decrease in the relative abundance of *Corynebacterium*, *Lactobacillus*, and other intestinal flora (Yu et al., 2017). In addition to this, we also found that *Corynebacterium* was significantly positively correlated with 5-HT, BDNF, and *Corynebacterium* showed significant positive correlations with N-Lauroylglycine, 7-Methylhypoxanthine, 5-Hydroxyindoleacetic acid, Indole-3-carboxaldehyde, Undecylenic acid, and 10,11-dihydro-20-trihydroxy-leukotriene B4. It suggests that these metabolites may be inextricably linked to the stimulation, synthesis, or increase of neurotransmitters in the brain of depressed mice. *Enterorhabdus* and *Desulfovibrio* were significantly negatively correlated with IL-6, TNF-α, and *Desulfovibrio* and *Enterorhabdus* were significantly correlated with the following metabolites LysoPC (18:2(9Z,12Z)/0:0), 5,8,11-Eicosatrienoic acid, 7-Methylhypoxanthine, 5-Hydroxyindoleacetic acid, and Indole-3-carboxaldehyde, suggesting that such metabolites could potentially play an important role in reducing the levels of pro-inflammatory factors. Previous studies have found that *Enterorhabdus* may be related to bile acid metabolism and that elevated *Enterorhabdus* affects the synthesis of beneficial bile acids (Wang et al., 2022; Xu W. et al., 2023); therefore, the antidepressant effect of Ziyan green tea may be partially mediated by reversing *Enterorhabdus* in CUMS mice. It is suggested that Ziyan green tea may act as an antidepressant through the gut-microbiota-brain axis or microbial-bile acid axis. In future studies, we will combine various research methods and perspectives, land on the pathways and targets of depression intervention, and further explore the mechanism of Ziyan green tea intervention in depression to alleviate CUMS-induced depression.

## 5. Conclusion

This study aimed to investigate the improvement of CUMS-induced depression-like symptoms by Ziyan green tea and its possible mechanism of action. Male mice were gavaged with Ziyan green tea. Depression-like behavior was measured by a series of behavioral tests and neurobiochemical factors (5-HT, DA, and BDNF) and pro-inflammatory factors (IL-6 and TNF-α) were measured in brain tissues, combined with Nissl pathological analysis and determination of gut microorganisms. Ziyan green tea intervention significantly attenuated CUMS-induced depression-like behaviors in mice. *Corynebacterium* showed a significant positive correlation with 5-HT and BDNF. *Faecalibaculum* and *Enterorhabdus* showed a significant negative correlation with IL-6, *Faecalibaculum* and *Desulfovibrio* showed a significant negative correlation with TNF-α. The microbial community diversity and richness of CUMS mice were significantly restored after Ziyan green tea intervention. Meanwhile, we performed UHPLC–MS/MS metabolomic analysis of mouse fecal samples to detect the content of relevant metabolites produced by intestinal microorganisms, and we identified a total of 20 differential metabolites such as Deoxycholic acid, LysoPC(18:2(9Z,12Z)/0:0), 12-Ketodeoxycholic acid, Cholic Acid, Deoxyinosine, 7-Methylhypoxanthine, 5,8,11-Eicosatrienoic acid, 5-Aminopentanoic acid, 10,11-dihydro-20-trihydroxy-leukotriene B4, 5-Hydroxyindoleacetic acid, L-Glutamate, Oxypurinol, Undecylenic acid, 7-Sulfocholic acid, Indole-3-carboxaldehyde, N-Acetylneuraminic Acid, L-Palmitoylcarnitine, N-Lauroylglycine, LysoPE(18:1(11Z)/0:0) and Uracil. And we found that *Alistipes* were significantly and negatively correlated with 7-Sulfocholic acid, 12-Ketodeoxycholic acid, and Cholic acid. In conclusion, our findings suggest that both low and high-dose interventions of Ziyan green tea have positive preventive effects on CUMS mice without dose dependence, partly because they mainly affect intestinal Purine Metabolism, Bile Acid Biosynthesis, and Cysteine Metabolism in CUMS mice, thus stimulating brain 5-HT, DA and BDNF, and decreasing the inflammatory factors IL-6, TNF-α, activate the composition of intestinal flora, improve the intestinal flora environment and thus promote the production of intestinal metabolites, which can be used for depression treatment. It is suggested that Ziyan green tea may achieve an antidepressant effect through the gut-microbiota-brain axis.

## Data availability statement

The datasets presented in this study can be found in online repositories. The names of the repository/repositories and accession number(s) can be found at: NCBI – PRJNA993842 (SRP449264).

## Ethics statement

The animal study was approved by Animal Ethics Committee of the Hunan Provincial Center for Drug Safety Evaluation and Research. The study was conducted in accordance with the local legislation and institutional requirements.

## Author contributions

WJ: Conceptualization, Data curation, Formal analysis, Software, Writing – original draft. QT: Investigation, Resources, Writing – original draft. YZ: Conceptualization, Methodology, Writing – original draft. YY: Conceptualization, Methodology, Writing – original draft. WW: Formal analysis, Funding acquisition, Writing – review & editing. WX: Conceptualization, Formal analysis, Funding acquisition, Methodology, Writing – review & editing.

## Funding

This work was supported by the Sichuan Province S&T Project (2023NZZJ0003, 2022ZYNC001); Hunan Agricultural Science and Technology Innovation Funds of China (2022CX33).

## Conflict of interest

YY was employed by Sichuan Yizhichun Tea Industry Co., Ltd.

The remaining authors declare that the research was conducted in the absence of any commercial or financial relationships that could be construed as a potential conflict of interest.

## Publisher’s note

All claims expressed in this article are solely those of the authors and do not necessarily represent those of their affiliated organizations, or those of the publisher, the editors and the reviewers. Any product that may be evaluated in this article, or claim that may be made by its manufacturer, is not guaranteed or endorsed by the publisher.

## Figures and Tables

**Table:** 

AA	Amino Acid
TPs	Tea Polyphenol
SS	Soluble Sugars
DL-C	Catechin
EC	Epicatechin
EGC	Epigallocatechin
ECG	Epicatechin gallate
GCG	Gallocatechin gallate
EGCG	Epigallocatechin gallate
GC	Gallocatechin
TB	Theobromine
THEO	Theophylline
CAF	Caffeine
TFs	Theaflavins
TRs	Thearubigins
TBs	Theabrownins
TAs	Total anthocyanins
UHPLC-QTOF/MS	Ultra-high-performance liquid chromatography quadrupole time-of-flight mass spectrometry.

## Footnotes

^1^http://qiime.org/scripts/assign_taxonomy.html

^2^https://www.metaboanalyst.ca/

^3^http://www.kegg.jp

^4^http://www.hmdb.ca

## References

[ref1] Aizawa E. Tsuji H. Asahara T. Takahashi T. Teraishi T. Yoshida S.. (2016). Possible association of bifidobacterium and lactobacillus in the gut microbiota of patients with major depressive disorder. J. Affect. Disord. 202, 254–257. doi: 10.1016/j.jad.2016.05.038, PMID: 27288567

[ref2] Axling U. Olsson C. Xu J. Fernandez C. Larsson S. Ström K.. (2012). Green tea powder and *lactobacillus plantarum* affect gut microbiota, lipid metabolism and inflammation in high-fat fed c57bl/6j mice. Nutr. Metab. 9:105. doi: 10.1186/1743-7075-9-105, PMID: 23181558 PMC3538623

[ref3] Bao H. Li H. Jia Y. Xiao Y. Luo S. Zhang D.. (2021). Ganoderic acid a exerted antidepressant-like action through fxr modulated nlrp3 inflammasome and synaptic activity. Biochem. Pharmacol. 188:114561. doi: 10.1016/j.bcp.2021.114561, PMID: 33857491

[ref4] Begley M. Gahan C. G. M. Hill C. (2005). The interaction between bacteria and bile. FEMS Microbiol. Rev. 29, 625–651. doi: 10.1016/j.femsre.2004.09.003 16102595

[ref5] Chang L. Wei Y. Hashimoto K. (2022). Brain–gut–microbiota axis in depression: a historical overview and future directions. Brain Res. Bull. 182, 44–56. doi: 10.1016/j.brainresbull.2022.02.004, PMID: 35151796

[ref6] Chen X. Eslamfam S. Fang L. Qiao S. Ma X. (2017). Maintenance of gastrointestinal glucose homeostasis by the gut-brain axis. Curr. Protein Pept. Sci. 18, 541–547. doi: 10.2174/1389203717666160627083604, PMID: 27356933

[ref8] Chow R. M. Issa M. (2019). Serotonin-norepinephrine reuptake inhibitors. Handb. Exp. Pharmacol. 250, 145–180. doi: 10.1007/164_2018_164 30838456

[ref9] Chunjing Y. Liqiang T. Changyin Y. Yang Y. Wei L. I. Xiaoqin T.. (2020). Anthocyanin-rich purple shoots tea cultivar ziyan. China Tea 42:8-11, 14.

[ref10] de la Garza A. Garza-Cuellar M. Silva-Hernandez I. Cardenas-Perez R. Reyes-Castro L. Zambrano E.. (2019). Maternal flavonoids intake reverts depression-like behaviour in rat female offspring. Nutrients 11:572. doi: 10.3390/nu11030572, PMID: 30866491 PMC6470771

[ref11] Doroszkiewicz J. Groblewska M. Mroczko B. (2021). The role of gut microbiota and gut–brain interplay in selected diseases of the central nervous system. Int. J. Mol. Sci. 22:10028. doi: 10.3390/ijms221810028, PMID: 34576191 PMC8471822

[ref12] Douglas G. M. Maffei V. J. Zaneveld J. R. Yurgel S. N. Brown J. R. Taylor C. M.. (2020). Picrust2 for prediction of metagenome functions. Nat. Biotechnol. 38, 685–688. doi: 10.1038/s41587-020-0548-6, PMID: 32483366 PMC7365738

[ref13] Dunlop B. W. Nemeroff C. B. (2007). The role of dopamine in the pathophysiology of depression. Arch. Gen. Psychiatry 64:327. doi: 10.1001/archpsyc.64.3.327 17339521

[ref14] Fan L. Peng Y. Wang J. Ma P. Zhao L. Li X. (2021). Total glycosides from stems of cistanche tubulosa alleviate depression-like behaviors: bidirectional interaction of the phytochemicals and gut microbiota. Phytomedicine 83:153471. doi: 10.1016/j.phymed.2021.153471, PMID: 33636477

[ref15] Goodwin F. K. Post R. M. (1983). 5-hydroxytryptamine and depression: a model for the interaction of normal variance with pathology. Br. J. Clin. Pharmacol. 15, 393S–405S. doi: 10.1111/j.1365-2125.1983.tb02130.x, PMID: 6190490 PMC1427648

[ref16] Gulbins A. Schumacher F. Becker K. A. Wilker B. Soddemann M. Boldrin F.. (2018). Antidepressants act by inducing autophagy controlled by sphingomyelin–ceramide. Mol. Psychiatry 23, 2324–2346. doi: 10.1038/s41380-018-0090-9, PMID: 30038230 PMC6294742

[ref17] Hara Y. (1997). Influence of tea catechins on the digestive tract. J. Cell. Biochem. Suppl. 27, 52–58. doi: 10.1002/(SICI)1097-4644(1997)27+<52::AID-JCB10>3.0.CO;2-N, PMID: 9591193

[ref18] Hashimoto K. (2022). Gut–microbiota–brain axis by bile acids in depression. Psychiatry Clin. Neurosci. 76:281. doi: 10.1111/pcn.13370, PMID: 35778785

[ref19] Herrman H. Patel V. Kieling C. Berk M. Buchweitz C. Cuijpers P.. (2022). Time for united action on depression: a lancet-world psychiatric association commission. Lancet 399, 957–1022. doi: 10.1016/S0140-6736(21)02141-3, PMID: 35180424

[ref20] Hu P. M. L. W. (2017). Genistein, a dietary soy isoflavone, exerts antidepressant-like effects in mice: involvement of serotonergic system. Neurochem. Int. 108, 426–435. doi: 10.1016/j.neuint.2017.06.002 28606822

[ref21] Jia W. Zhao Y. Liao S. Li P. Zou Y. Chen S.. (2022). Dynamic changes in the diversity and function of bacterial community during black tea processing. Food Res. Int. 161:111856. doi: 10.1016/j.foodres.2022.111856, PMID: 36192903

[ref22] Jinhua C. Bin T. Yushun G. Jianan H. Zhonghua L. (2015). Effect of black tea on regulating serum lipid in mice fed with a high-fat diet. J. Tea Sci. 4, 384–396. doi: 10.3969/j.issn.1000-369X.2015.04.017

[ref23] Kandola A. Ashdown-Franks G. Hendrikse J. Sabiston C. M. Stubbs B. (2019). Physical activity and depression towards understanding the antidepressant mechanisms of physical activity. Neurosci. Biobehav. Rev. 107, 525–539. doi: 10.1016/j.neubiorev.2019.09.040, PMID: 31586447

[ref24] Koh A. Bäckhed F. (2020). From association to causality: the role of the gut microbiota and its functional products on host metabolism. Mol. Cell 78, 584–596. doi: 10.1016/j.molcel.2020.03.005 32234490

[ref25] Köhler C. A. Freitas T. H. Maes M. de Andrade N. Q. Liu C. S. Fernandes B. S.. (2017). Peripheral cytokine and chemokine alterations in depression: a meta-analysis of 82 studies. Acta Psychiatr. Scand. 135, 373–387. doi: 10.1111/acps.12698, PMID: 28122130

[ref26] Krikorian R. Shidler M. D. Nash T. A. Kalt W. Vinqvist-Tymchuk M. R. Shukitt-Hale B.. (2010). Blueberry supplementation improves memory in older adults. J. Agric. Food Chem. 58, 3996–4000. doi: 10.1021/jf9029332, PMID: 20047325 PMC2850944

[ref27] Kumar B. Arora V. Kuhad A. Chopra K. (2012). *Vaccinium myrtillus* ameliorates unpredictable chronic mild stress induced depression: possible involvement of nitric oxide pathway. Phytother. Res. 26, 488–497. doi: 10.1002/ptr.3584, PMID: 22488796

[ref28] Lach G. Schellekens H. Dinan T. G. Cryan J. F. (2018). Anxiety, depression, and the microbiome: a role for gut peptides. Neurotherapeutics 15, 36–59. doi: 10.1007/s13311-017-0585-0, PMID: 29134359 PMC5794698

[ref29] Lee J. Durst R. W. Wrolstad R. E. (2005). Determination of total monomeric anthocyanin pigment content of fruit juices, beverages, natural colorants, andwines by the PH differential method: collaborative study. J. AOAC Int. 88, 1269–1278. doi: 10.1093/jaoac/88.5.1269, PMID: 16385975

[ref30] Li H. Shi J. Zhao L. Guan J. Liu F. Huo G.. (2021). *Lactobacillus plantarum* klds1.0344 and *lactobacillus acidophilus* klds1.0901 mixture prevents chronic alcoholic liver injury in mice by protecting the intestinal barrier and regulating gut microbiota and liver-related pathways. J. Agric. Food Chem. 69, 183–197. doi: 10.1021/acs.jafc.0c06346, PMID: 33353302

[ref31] Li H. Xiang Y. Zhu Z. Wang W. Jiang Z. Zhao M.. (2021). Rifaximin-mediated gut microbiota regulation modulates the function of microglia and protects against cums-induced depression-like behaviors in adolescent rat. J. Neuroinflammation 18:254. doi: 10.1186/s12974-021-02303-y, PMID: 34736493 PMC8567657

[ref32] Liu Y. Hu J. Li M. M. Zhao G. (2023). Effects of taurine on rumen fermentation, nutrient digestion, rumen bacterial community and metabolomics and nitrogen metabolism in beef steers. J. Sci. Food Agric. 103, 3414–3426. doi: 10.1002/jsfa.12474, PMID: 36710505

[ref33] Liu D. Huang J. Luo Y. Wen B. Wu W. Zeng H.. (2019). Fuzhuan brick tea attenuates high-fat diet-induced obesity and associated metabolic disorders by shaping gut microbiota. J. Agric. Food Chem. 67, 13589–13604. doi: 10.1021/acs.jafc.9b05833, PMID: 31735025

[ref34] Liu Y. Wu Z. Cheng L. Zhang X. Yang H. (2021). The role of the intestinal microbiota in the pathogenesis of host depression and mechanism of tps relieving depression. Food Funct. 12, 7651–7663. doi: 10.1039/d1fo01091c, PMID: 34286799

[ref35] Liu Y. Zhang L. Wang X. Wang Z. Zhang J. Jiang R.. (2016). Similar fecal microbiota signatures in patients with diarrhea-predominant irritable bowel syndrome and patients with depression. Clin. Gastroenterol. Hepatol. 14, 1602–1611.e5. doi: 10.1016/j.cgh.2016.05.033, PMID: 27266978

[ref36] Liu C. Zhao D. Ma W. Guo Y. Wang A. Wang Q.. (2016). Denitrifying sulfide removal process on high-salinity wastewaters in the presence of halomonas sp. Appl. Microbiol. Biotechnol. 100, 1421–1426. doi: 10.1007/s00253-015-7039-6, PMID: 26454867

[ref37] Lourenço C. Kelly D. Cantillon J. Cauchi M. Yon M. A. Bentley L.. (2019). Monitoring type 2 diabetes from volatile faecal metabolome in cushing’s syndrome and single afmid mouse models via a longitudinal study. Sci. Rep. 9:18779. doi: 10.1038/s41598-019-55339-9, PMID: 31827172 PMC6906526

[ref38] Lucidi L. Pettorruso M. Vellante F. Di Carlo F. Ceci F. Santovito M. C.. (2021). Gut microbiota and bipolar disorder: an overview on a novel biomarker for diagnosis and treatment. Int. J. Mol. Sci. 22:3723. doi: 10.3390/ijms22073723, PMID: 33918462 PMC8038247

[ref39] Luo X. Huang X. Luo Z. Wang Z. He G. Tan Y.. (2021). Electromagnetic field exposure-induced depression features could be alleviated by heat acclimation based on remodeling the gut microbiota. Ecotoxicol. Environ. Saf. 228:112980. doi: 10.1016/j.ecoenv.2021.112980, PMID: 34794024

[ref40] Mayer E. A. Tillisch K. Gupta A. (2015). Gut/brain axis and the microbiota. J. Clin. Invest. 125, 926–938. doi: 10.1172/JCI76304, PMID: 25689247 PMC4362231

[ref7] Ministry of Health of the People’s Republic of China. (2003). Technical standards for testing assessment of health food. [Z]. 2003, 35–36.

[ref41] Nabavi S. M. Daglia M. Braidy N. Nabavi S. F. (2017). Natural products, micronutrients, and nutraceuticals for the treatment of depression: a short review. Nutr. Neurosci. 20, 180–194. doi: 10.1080/1028415X.2015.1103461, PMID: 26613119

[ref42] NgT. P.GaoQ.GweeX.ChuaD. (2021). Tea consumption and depression from follow up in the Singapore longitudinal ageing study. J. Nutr. Health Aging 25, 295–301. doi: 10.1007/s12603-020-1526-x, PMID: 33575719

[ref43] Nukina H. Sudo N. Aiba Y. Oyama N. Koga Y. Kubo C. (2001). Restraint stress elevates the plasma interleukin-6 levels in germ-free mice. J. Neuroimmunol. 115, 46–52. doi: 10.1016/S0165-5728(01)00260-0, PMID: 11282153

[ref44] Parker B. J. Wearsch P. A. Veloo A. C. M. Rodriguez-Palacios A. (2020). The genus alistipes: gut bacteria with emerging implications to inflammation, cancer, and mental health. Front. Immunol. 11:906. doi: 10.3389/fimmu.2020.00906, PMID: 32582143 PMC7296073

[ref45] Pervin M. Unno K. Ohishi T. Tanabe H. Miyoshi N. Nakamura Y. (2018). Beneficial effects of green tea catechins on neurodegenerative diseases. Molecules 23:1297. doi: 10.3390/molecules23061297, PMID: 29843466 PMC6099654

[ref46] Postal M. Appenzeller S. (2015). The importance of cytokines and autoantibodies in depression. Autoimmun. Rev. 14, 30–35. doi: 10.1016/j.autrev.2014.09.001, PMID: 25242344

[ref47] Qiao Y. Zhao J. Li C. Zhang M. Wei L. Zhang X.. (2020). Effect of combined chronic predictable and unpredictable stress on depression-like symptoms in mice. Ann. Transl. Med. 8:942. doi: 10.21037/atm-20-5168, PMID: 32953742 PMC7475446

[ref48] Ridlon J. M. Kang D. J. Hylemon P. B. Bajaj J. S. (2014). Bile acids and the gut microbiome. Curr. Opin. Gastroenterol. 30, 332–338. doi: 10.1097/MOG.0000000000000057, PMID: 24625896 PMC4215539

[ref49] Rosa J. M. Formolo D. A. Yu J. Lee T. H. Yau S. (2022). The role of microrna and microbiota in depression and anxiety. Front. Behav. Neurosci. 16:8258. doi: 10.3389/fnbeh.2022.828258, PMID: 35299696 PMC8921933

[ref50] Rothenberg D. O. N. Zhang L. (2019). Mechanisms underlying the anti-depressive effects of regular tea consumption. Nutrients 11:1361. doi: 10.3390/nu11061361, PMID: 31212946 PMC6627400

[ref51] Shao J. Wei Y. Wei X. (2022). A comprehensive review on bioavailability, safety and antidepressant potential of natural bioactive components from tea. Food Res. Int. 158:111540. doi: 10.1016/j.foodres.2022.111540, PMID: 35840236

[ref52] Sharma H. R. Thakur M. K. (2015). Correlation of erα/erβ expression with dendritic and behavioural changes in cums mice. Physiol. Behav. 145, 71–83. doi: 10.1016/j.physbeh.2015.03.041, PMID: 25837835

[ref53] Shen X. Li X. Jia C. Li J. Chen S. Gao B.. (2023). Hplc-ms-based untargeted metabolomic analysis of differential plasma metabolites and their associated metabolic pathways in reproductively anosmic black porgy, *acanthopagrus schlegelii*. Compar. Biochem. Physiol. D Genom. Proteom. 46:101071. doi: 10.1016/j.cbd.2023.101071, PMID: 36931130

[ref54] Shen W. Tao Y. Zheng F. Zhou H. Wu H. Shi H.. (2023). The alteration of gut microbiota in venlafaxine-ameliorated chronic unpredictable mild stress-induced depression in mice. Behav. Brain Res. 446:114399. doi: 10.1016/j.bbr.2023.114399, PMID: 36963638

[ref55] Siddiqui I. A. Afaq F. Adhami V. M. Ahmad N. (2004). Antioxidants of the beverage tea in promotion of human health. Antioxid. Redox Signal. 6, 571–582. doi: 10.1089/152308604773934323 15130283

[ref56] Song J. Zhou N. Ma W. Gu X. Chen B. Zeng Y.. (2019). Modulation of gut microbiota by chlorogenic acid pretreatment on rats with adrenocorticotropic hormone induced depression-like behavior. Food Funct. 10, 2947–2957. doi: 10.1039/C8FO02599A, PMID: 31073553

[ref57] Spohr L. Luduvico K. P. Soares M. S. P. Bona N. P. Oliveira P. S. de Mello J. E.. (2022). Blueberry extract as a potential pharmacological tool for preventing depressive-like behavior and neurochemical dysfunctions in mice exposed to lipopolysaccharide. Nutr. Neurosci. 25, 857–870. doi: 10.1080/1028415X.2020.1819104, PMID: 32954970

[ref58] Sun J. Wang F. Hu X. Yang C. Xu H. Yao Y.. (2018). *Clostridium butyricum* attenuates chronic unpredictable mild stress-induced depressive-like behavior in mice via the gut-brain axis. J. Agric. Food Chem. 66, 8415–8421. doi: 10.1021/acs.jafc.8b02462, PMID: 30040410

[ref59] Sushma G. Vaidya B. Sharma S. Devabattula G. Bishnoi M. Kondepudi K. K.. (2023). *Bifidobacterium breve* bif11 supplementation improves depression-related neurobehavioural and neuroinflammatory changes in the mouse. Neuropharmacology 229:109480. doi: 10.1016/j.neuropharm.2023.109480, PMID: 36868402

[ref60] Takebayashi N. Maeshima H. Baba H. Nakano Y. Satomura E. Kita Y.. (2012). Duration of last depressive episode may influence serum bdnf levels in remitted patients with major depression. Depress. Anxiety 29, 775–779. doi: 10.1002/da.21933, PMID: 22447660

[ref61] Tian P. Chen Y. Zhu H. Wang L. Qian X. Zou R.. (2022). *Bifidobacterium breve* ccfm1025 attenuates major depression disorder via regulating gut microbiome and tryptophan metabolism: a randomized clinical trial. Brain Behav. Immun. 100, 233–241. doi: 10.1016/j.bbi.2021.11.023, PMID: 34875345

[ref62] Tian P. O'Riordan K. J. Lee Y. K. Wang G. Zhao J. Zhang H.. (2020). Towards a psychobiotic therapy for depression: *bifidobacterium breve* ccfm1025 reverses chronic stress-induced depressive symptoms and gut microbial abnormalities in mice. Neurobiol. Stress. 12:100216. doi: 10.1016/j.ynstr.2020.100216, PMID: 32258258 PMC7109524

[ref63] Wang W. Qin X. Wang R. Xu J. Wu H. Khalid A.. (2020). Ezh2 is involved in vulnerability to neuroinflammation and depression-like behaviors induced by chronic stress in different aged mice. J. Affect. Disord. 272, 452–464. doi: 10.1016/j.jad.2020.03.154, PMID: 32553389

[ref64] Wang S. Wang S. Wang X. Xu Y. Zhang X. Han Y.. (2022). Effects of icariin on modulating gut microbiota and regulating metabolite alterations to prevent bone loss in ovariectomized rat model. Front. Endocrinol. 13:4849. doi: 10.3389/fendo.2022.874849, PMID: 35399950 PMC8988140

[ref65] Wenliang W. Liu Z. Lin Y. Huang J. A. Zuo G. Cuiqin T.. (2018). Alleviative effects of aged liupao tea on lipid metabolism and liver injury in hyperlipidemic mice. J. Tea Sci. 38, 430–438. doi: 10.13305/j.cnki.jts.2018.04.012

[ref66] Xiong Z. Yang J. Huang Y. Zhang K. Bo Y. Lu X.. (2016). Serum metabonomics study of anti-depressive effect of xiao-chai-hu-tang on rat model of chronic unpredictable mild stress. J. Chromatogr. B 1029-1030, 28–35. doi: 10.1016/j.jchromb.2016.06.044, PMID: 27398633

[ref67] Xu K. Gao X. Xia G. Chen M. Zeng N. Wang S.. (2021). Rapid gut dysbiosis induced by stroke exacerbates brain infarction in turn. Gut 70, 1486–1494. doi: 10.1136/gutjnl-2020-323263, PMID: 33558272

[ref68] Xu W. Kong Y. Zhang T. Gong Z. Xiao W. (2023). L-theanine regulates lipid metabolism by modulating gut microbiota and bile acid metabolism. J. Sci. Food Agric. 103, 1283–1293. doi: 10.1002/jsfa.12222 36087337

[ref69] Xu H. Xie S. Liu T. Zhou X. Feng Z. He X. (2023). Microbiota alteration of chinese young male adults with high-status negative cognitive processing bias. Front. Microbiol. 14:989162. doi: 10.3389/fmicb.2023.989162, PMID: 36937259 PMC10015002

[ref70] Yang F. Feng B. Niu Y. J. Hu C. Y. Meng Y. H. (2021). Fu instant tea ameliorates fatty liver by improving microbiota dysbiosis and elevating short-chain fatty acids in the intestine of mice fed a high-fat diet. Food Biosci. 42:101207. doi: 10.1016/j.fbio.2021.101207

[ref71] Yang C. Hu Z. Lu M. Li P. Tan J. Chen M.. (2018). Application of metabolomics profiling in the analysis of metabolites and taste quality in different subtypes of white tea. Food Res. Int. 106, 909–919. doi: 10.1016/j.foodres.2018.01.069, PMID: 29580004

[ref72] Yang J. Zheng P. Li Y. Wu J. Tan X. Zhou J.. (2020). Landscapes of bacterial and metabolic signatures and their interaction in major depressive disorders. Sci. Adv. 6:8555. doi: 10.1126/sciadv.aba8555, PMID: 33268363 PMC7710361

[ref73] Yao Y. Chen H. Chen L. Ju S. Yang H. Zeng Y.. (2021). Type of tea consumption and depressive symptoms in chinese older adults. BMC Geriatr. 21:331. doi: 10.1186/s12877-021-02203-z, PMID: 34030654 PMC8142291

[ref74] Yu M. Jia H. Zhou C. Yang Y. Zhao Y. Yang M. (2017). Variations in gut microbiota and fecal metabolic phenotype associated with depression by 16s rrna gene sequencing and lc/ms-based metabolomics. J. Pharm. Biomed. Anal. 138, 231–239. doi: 10.1016/j.jpba.2017.02.008, PMID: 28219800

[ref75] Zhang Y. Huang J. Xiong Y. Zhang X. Lin Y. Liu Z. (2022). Jasmine tea attenuates chronic unpredictable mild stress-induced depressive-like behavior in rats via the gut-brain axis. Nutrients 14:99. doi: 10.3390/nu14010099, PMID: 35010973 PMC8746588

[ref76] Zhang M. Li A. Yang Q. Li J. Wang L. Liu X.. (2021). Beneficial effect of alkaloids from sophora alopecuroides l. on cums-induced depression model mice via modulating gut microbiota. Front. Cell. Infect. Microbiol. 11:5159. doi: 10.3389/fcimb.2021.665159, PMID: 33954123 PMC8089385

[ref77] Zhang M. Tao X. Pan R. Wang L. Li C. Zhou Y.. (2020). Antidepressant-like effects of cajaninstilbene acid and its related mechanisms in mice. Fitoterapia 141:104450. doi: 10.1016/j.fitote.2019.104450, PMID: 31837410

[ref78] Zhang X. Tian J. Liu H. Xue-Mei Q. (2017). Progress of new antidepressant drugs development. Zhongguo Zhong Yao Za Zhi 42, 29–33. doi: 10.19540/j.cnki.cjcmm.20161222.056, PMID: 28945021

[ref79] Zhang X. Zhu X. Sun Y. Hu B. Sun Y. Jabbar S.. (2013). Fermentation *in vitro* of egcg, gcg and egcg3 me isolated from oolong tea by human intestinal microbiota. Food Res. Int. 54, 1589–1595. doi: 10.1016/j.foodres.2013.10.005

[ref80] Zhao W. Sun G. (2010). Conversion of drug dosage between different species of experimental animals. Chinese J. Anim. Husbandry Vet. Med., 52–53. doi: 10.3969/J.ISSN.1671-6027.2010.05.032

[ref81] Zhou J. Tang L. Shen C. Wang J. (2020). Green tea polyphenols boost gut-microbiota-dependent mitochondrial tca and urea cycles in Sprague–dawley rats. J. Nutr. Biochem. 81:108395. doi: 10.1016/j.jnutbio.2020.108395, PMID: 32388254

[ref82] Zhu W. Shi H. Wei Y. Wang S. Sun C. Ding Z.. (2012). Green tea polyphenols produce antidepressant-like effects in adult mice. Pharmacol. Res. 65, 74–80. doi: 10.1016/j.phrs.2011.09.007, PMID: 21964320

