# Supplementary material for: Investigating the antidepressant effect of Ziyan green tea on chronic unpredictable mild stress mice through fecal metabolomics
Source: Front Microbiol. 2023 Aug 24;14:1256142. doi: 10.3389/fmicb.2023.1256142 (PMC10483239; doi:10.3389/fmicb.2023.1256142)
Supplement: Supplementary file 1 [file Data_Sheet_1.pdf]

Investigating the antidepressant effect of Ziyan green tea on CUMS mice through fecal metabolomics

Wenbao Jia[1],[2], Qian Tang[1], Yao Zou[1], Yang Yang[3], Wenliang Wu[2,*], Wei Xu[1,*]

[1] College of Horticulture, Tea Refining and Innovation Key Laboratory of Sichuan Province, Sichuan Agricultural University, Chengdu 611130, China

[2] Tea Research Institute, Hunan Academy of Agricultural Sciences, Changsha, Hunan, China, Changsha 410145, China

[3] Sichuan Yizhichun Tea Industry Co., Ltd., Muchuan, Sichuan, 614500, China

* Corresponding authors:

Wenliang Wu: wwlvip8@163.com

Wei Xu: xuweianti@sicau.edu.cn

(UHPLC-MS/MS) analysis

Chromatographic conditions：

2μL of sample was separated by HSS T3 column (100 mm×2.1 mm i.d., 1.8μm) and then entered into mass spectrometry detection.The mobile phases consisted of 0.1% formic acid in water:acetonitrile (95:5, v/v) (solvent A) and 0.1% formic acid in acetonitrile:isopropanol:water (47.5:47.5:5, v/v)(solvent B). The solvent gradient changed according to the following conditions: from 0 to 3.5 min, 0% B to 24.5% B (0.4 mL/min); from 3.5 to 5 min, 24.5% B to 65% B (0.4 mL/min); from 5 to 5.5 min, 65% B to 100% B (0.4 mL/min); from 5.5to 7.4 min, 100% B to 100% B (0.4 mL/min to 0.6 mL/min); from 7.4 to 7.6 min, 100% B to 51.5% B (0.6 mL/min); from 7.6 to 7.8 min, 51.5% B to 0% B (0.6 mL/min to 0.5 mL/min); from 7.8 to 9 min, 0% B to 0% B (0.5 mL/min to 0.4 mL/min);from 9 to 10 min, 0% B to 0% B (0.4 mL/min) for equilibrating the systems. The sample injection volume was 2 μL and the flow rate was set to 0.4 mL/min. The column temperature was maintained at 40℃. During the period of analysis, all these samples were stored at 4℃.

MS conditions:

The mass spectrometric data was collected using a Thermo UHPLC -Q Exactive HF-X Mass Spectrometer equipped with an electrospray ionization (ESI) source operating in either positive or negative ion mode. The optimal conditions were set as followed: heater temperature, 425℃ ; Capillary temperature, 325℃; sheath gas flow rate, 50 arb; Aux gas flow rate, 13 arb; ion-spray voltage floating (ISVF), -3500V in negative mode and 3500V in positive mode, respectively; Normalized collision energy , 20-40-60V rolling for MS/MS. Full MS resolution was 60000, and MS/MS resolution was 7500. Data acquisition was performed with the Data Dependent Acquisition (DDA) mode. The detection was carried out over a mass range of 70-1050 m/z.

Table S1 Relevant secondary metabolite contents in aqueous extracts of Ziyan green tea

Constituent	Ziyan green tea (Mean±SD)
Amino acid (%)	9.90±0.10
Tea polyphenol (%)	49.39±1.54
Soluble sugars (%)	6.59±0.29
GC (%)	0.32±0.01
EGC (%)	5.12±0.1
DL-C (%)	0.29±0.01
EC (%)	0.75±0.03
EGCG (%)	15.68±0.3
GCG (%)	1.17±0.02
ECG (%)	2.27±0.06
Total catechins (%)	25.25±0.74
Theobromine (%)	0.57±0.02
Theophylline (%)	0.03±0.00
Caffeine (%)	6.68±0.15
Theaflavins (%)	0.39±0.00
Thearubigins (%)	6.77±0.11
Theabrownins (%)	6.70±0.24
Total anthocyanins (%)	5.55±0.15

Table S2 Study on fecal biomarkers of Ziyan green tea in mice with CUMS depression.

	Total	Expected	Hits	Raw p	-log$_{10}$(p)	Holm adjust	FDR	Impact
Purine Metabolism	63	0.62687	3	0.02053	1.6876	1	1	0.048378
Bile Acid Biosynthesis	60	0.59701	2	0.11584	0.93614	1	1	0.0090792
Cysteine Metabolism	24	0.23881	1	0.21558	0.6664	1	1	0
Beta-Alanine Metabolism	26	0.25871	1	0.2315	0.63546	1	1	0
Amino Sugar Metabolism	31	0.30846	1	0.27003	0.56859	1	1	0.086538
Fatty Acid Metabolism	40	0.39801	1	0.33503	0.47491	1	1	0
Pyrimidine Metabolism	54	0.53731	1	0.42584	0.37075	1	1	0.035536
Tryptophan Metabolism	55	0.54726	1	0.43188	0.36464	1	1	0.037963

[a] Total indicates the total number of metabolites contained in the pathway, hits indicates that the pathway contains differential metabolites, Raw P values were calculated from enrichment analysis; impact values were calculated from pathway topology analysis, pathway impact indicates the value of the horizontal coordinate of Figure 7B, and the vertical axis is the -$\log_{10}(p)$ value, which is a weighting calculation based on topological analysis (It is calculated adding up the importance measures of each of the matched metabolites and then dividing by the sum of the importance measures of all metabolites in each pathway.)

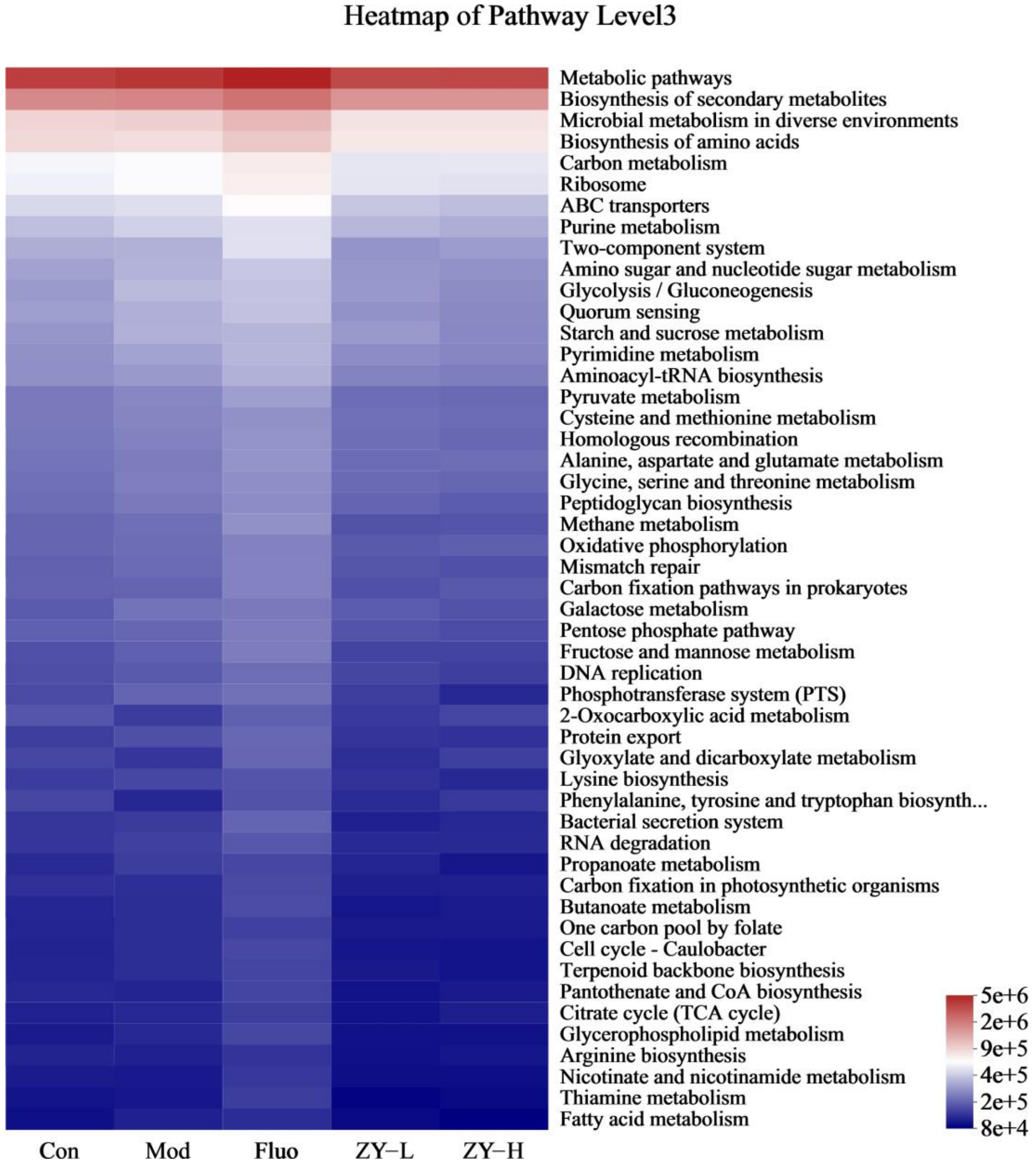

Figure S1. KEGG metabolic pathways enriched in the gut microbiota of CUMS mice

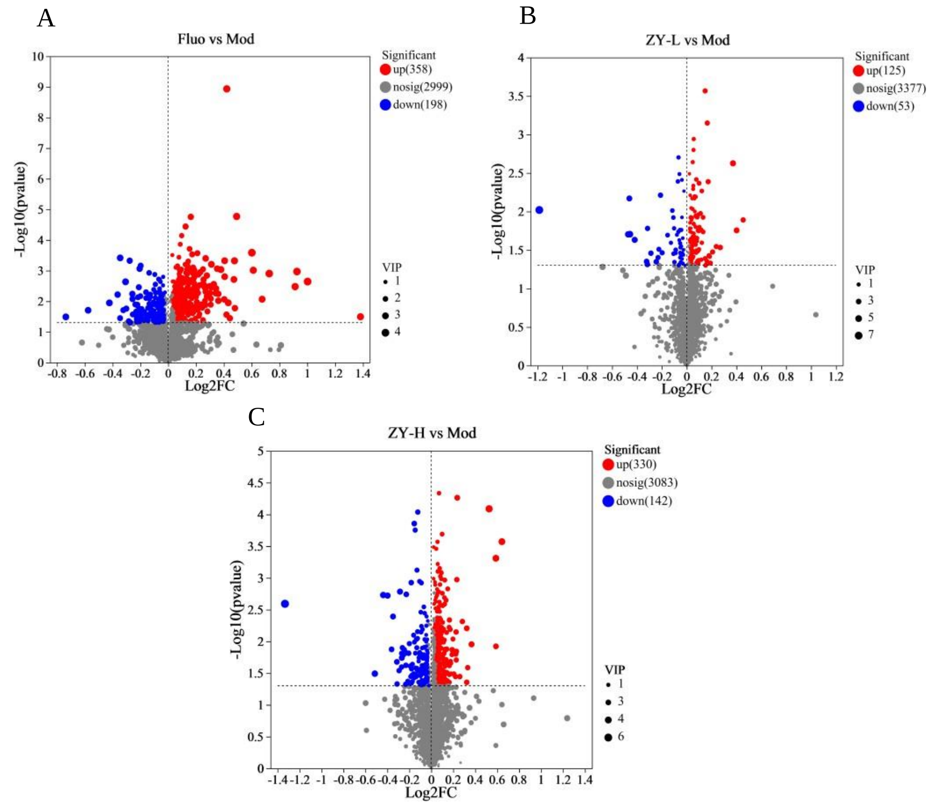

Figure S2 Volcano map for differential metabolite screening. A. Fluo vs Mod; B. ZY-L vs Mod; C. ZY-H vs Mod.
